# Combining in vitro assays and mathematical modelling to study developmental neurotoxicity induced by chemical mixtures

**DOI:** 10.1016/j.reprotox.2021.08.007

**Published:** 2021-10

**Authors:** Francesca Pistollato, Donatella Carpi, Emilio Mendoza-de Gyves, Alicia Paini, Stephanie K. Bopp, Andrew Worth, Anna Bal-Price

**Affiliations:** European Commission, Joint Research Centre (JRC), Ispra, Italy

**Keywords:** Mixture risk assessment, Combined toxicity, Adverse outcome pathway, Human induced pluripotent stem cells, Neuronal differentiation, Mathematical modelling, Children’s health

## Abstract

Prenatal and postnatal co-exposure to multiple chemicals at the same time may have deleterious effects on the developing nervous system. We previously showed that chemicals acting through similar mode of action (MoA) and grouped based on perturbation of brain derived neurotrophic factor (BDNF), induced greater neurotoxic effects on human induced pluripotent stem cell (hiPSC)-derived neurons and astrocytes compared to chemicals with dissimilar MoA. Here we assessed the effects of repeated dose (14 days) treatments with mixtures containing the six chemicals tested in our previous study (Bisphenol A, Chlorpyrifos, Lead(II) chloride, Methylmercury chloride, PCB138 and Valproic acid) along with 2,2′4,4′-tetrabromodiphenyl ether (BDE47), Ethanol, Vinclozolin and 2,3,7,8-tetrachlorodibenzo-p-dioxin (TCDD)), on hiPSC-derived neural stem cells undergoing differentiation toward mixed neurons/astrocytes up to 21 days. Similar MoA chemicals in mixtures caused an increase of BDNF levels and neurite outgrowth, and a decrease of synapse formation, which led to inhibition of electrical activity. Perturbations of these endpoints are described as common key events in adverse outcome pathways (AOPs) specific for DNT. When compared with mixtures tested in our previous study, adding similarly acting chemicals (BDE47 and EtOH) to the mixture resulted in a stronger downregulation of synapses. A synergistic effect on some synaptogenesis-related features (PSD95 in particular) was hypothesized upon treatment with tested mixtures, as indicated by mathematical modelling. Our findings confirm that the use of human iPSC-derived mixed neuronal/glial models applied to a battery of in vitro assays anchored to key events in DNT AOP networks, combined with mathematical modelling, is a suitable testing strategy to assess in vitro DNT induced by chemical mixtures.

## Highlights

• Mixtures of similar MoA chemicals increased BDNF levels and neurite outgrowth.
• Mixtures of similar MoA chemicals decreased synapse formation and electrical activity.
• Synergistic effects on synaptogenesis features was predicted by mathematical modelling.
• Some of the DNT effects observed in hiPSC-neurons/astrocytes recapitulate ASD features.
• In vitro assays combined with mathematical modelling enable DNT testing of chemical mixtures.

## 1 Introduction

Human beings at any stage of their life, including pregnancy, are exposed to multiple substances at the same time [1]. Epidemiological studies have shown that various classes of chemicals can be found in different types of biological samples, including breast milk [2], cord blood [3], children’s blood [4], urine [5], and hair [6]. For this reason, the implementation of testing strategies suitable for mixture risk assessment (MRA) and to assess the effects of chemical mixtures on human health-related endpoints, has been intensively debated over the last decade.

Brain development is a highly complex process taking place both during the gestational and post-natal phases, and characterized by multiple biological processes, spanning from neural progenitor cell commitment, proliferation, migration, neuronal and glial cell differentiation, neurite outgrowth, synaptogenesis, and neuronal network formation and function, which are regulated by several signalling pathways, such as Wnt, Sonic hedgehog, Notch, BMP, and ERK-CREB-BDNF [7,8].

Perturbations of some of these neurodevelopmental processes (i.e., brain derived neurotrophic factor (BDNF) levels, synaptogenesis and neuronal network formation and function) are described as common key events (CKEs) in an adverse outcome pathway (AOP) network for human neurotoxicity [9] that leads to the same adverse outcome (AO) (i.e., learning and memory impairment in children) [[10], [11], [12], [13], [14], [15], [16], [17], [18], [19], [20], [21], [22], [23], [24], [25], [26], [27]].

Nowadays, it is possible to investigate these CKEs using batteries of in vitro assays based on the use of non-animal models, such as human induced pluripotent stem cell (hiPSC)-derived neuronal and glial cultures, which have been proven reliable test systems to assess DNT in vitro [[28], [29], [30], [31], [32]]. Such in vitro models combined with assays anchored to CKEs of DNT AOPs can also be applied for MRA, since these CKEs are triggered by different MIEs, and thus different classes of chemicals [31,32].

This approach enables a mechanistic understanding about the possible impact of chemical mixtures on neurodevelopmental processes, which is not captured by the current in vivo DNT testing following either the OECD TG 426 for DNT, or the OECD TG 443 (extended one generation reproductive toxicity study) [33], the latter including also pre-mating exposure and cognitive learning and memory tests triggered only on the basis of available data. Opposite to human-based in vitro tests covering different neurodevelopmental endpoints, these in vivo studies are limited by extrapolation uncertainties relative to interspecies differences in brain development, and by their general lack of mechanistic information.

The regulatory basis for this work is described in the 2013″Scientific Opinion" of EFSA Plant Protection Products and their Residues (PPR) Panel on the relevance of dissimilar mode of action (MoA) for pesticide residues in food" [34], and the more recent guidance on risk assessment of combined exposure to multiple chemicals [35]. We have previously shown that clustering well-known DNT chemicals in mixtures on the basis of their MoA into 'similar' and 'dissimilar', but still leading to the same AO, is a suitable strategy to investigate in vitro MRA in relation to DNT and evaluate, by means of mathematical modelling, whether mixture effects are greater than (or similar to) those triggered by the most potent single chemicals [31,32]. Following the principles described in the EFSA documents [34,35] on grouping of chemicals on the basis of their mechanism of action, chemicals working through ‘similar MoA’ should share at least one common mechanism (in this case, alteration of BDNF levels leading to (or associated with) alterations of synaptogenesis), whilst chemicals characterized by ‘dissimilar MoA’ work through multiple mechanisms, but in our case, not directly linked to alterations of BDNF levels. Both chemical groups lead to the same adverse outcome, i.e., impairment of learning and memory in children.

In our previous study, we created three different mixture compositions using chemicals belonging to different classes: a mixture with 3-similar MoA chemicals (with Lead(II) chloride (Lead, a heavy metal), Chlorpyrifos (CPF, a pesticide), and Bisphenol A (BPA, an industrial chemical)); a mixture with 3-dissimilar MoA chemicals (containing Methylmercury(II) chloride (Methyl-Hg, a heavy metal), Valproic acid (VA, an antiepileptic drug), and PCB138, a chlorinated persistent organic pollutant); and a mixture containing all these six chemicals together. These chemicals have been shown to be present in human biological samples, such as maternal and infants’ blood, urine, cord blood, breast milk and hair [2,31,[36], [37], [38], [39]], and have been associated with cognitive impairments in children, as described in several epidemiological studies [[40], [41], [42], [43], [44], [45], [46], [47], [48], [49], [50]].

In this follow-up study, we assessed the effects of repeated dose (14d) treatments with more complex chemical mixtures characterized by the presence of either 5 or 10 chemicals. For this purpose, four additional chemicals were selected on top of the six chemicals previously tested based on their established impact on BDNF levels, synaptogenesis and their association with cognitive impairment, as summarized in Supplementary Table S1. The four additional chemicals were: 2,2′4,4′-tetrabromodiphenyl ether (BDE47, a brominated persistent organic pollutant), Ethanol (EtOH, an industrial chemical), Vinclozolin (Vincl, a fungicide), and 2,3,7,8-tetrachlorodibenzo-p-dioxin (TCDD, a chlorinated persistent organic pollutant). Following the same selection criteria adopted in our previous study, these four new chemicals were selected based on: (i) their proven association with cognitive/learning and memory impairment in children described in epidemiological studies; (ii) their mode of action, considering their effects on synaptogenesis (possibly occurring as consequence of BDNF signalling alteration); (iii) their heterogeneous chemical classification; and (iv) their detection in human samples (Supplementary Table S1). The resulting mixtures were composed as follows: a mixture combining five similar MoA chemicals (5-Sim: BPA, CPF, Lead, BDE47 and EtOH), a mixture with five dissimilar MoA chemicals (5-Diss: Methyl-Hg, PCB138, VA, Vincl and TCDD), and a mixture containing all 10 chemicals (10-All).

The effects of these 10 chemicals combined in different mixtures were compared against previously studied DNT effects induced by six compounds and their mixtures, to evaluate whether the presence of the four additional chemicals made a difference. Furthermore, mathematical modelling was used to identify combined effects (i.e., additive effects and any deviations from additivity, i.e., synergism) of chemicals in mixtures.

## 2 Materials and methods

### 2.1 Differentiation of human induced pluripotent stem cell (hiPSC)-derived neural stem cells (NSCs) into neurons and astrocytes

Neural stem cells (NSCs), originally derived from IMR90-hiPSCs (directly reprogrammed and kindly provided by Prof. Marc Peschanski, I-Stem, France), were differentiated into a mixed culture of neurons and astrocytes, as previously described [31,51]. In brief, NSCs were passaged, plated onto reduced growth factor matrigel-coated 96 well poly-d-lysine-coated-plates (Corning) at a density of 7000 cells/well (i.e., 21.000 cells/cm^2^, 150 μL/well), and differentiated for 21d in vitro (DIV). For analysis of electrical activity, upon passaging, 50.000 cells were spot-plated onto 24-well multielectrode array (MEA) plates (see section below). After 21 DIV, a heterogeneous culture of neurons (glutamatergic (35–42 %), GABAergic (15–20 %), and dopaminergic (13–20 %)), astrocytes (18–24 %), with a remaining proportion of nestin^+^ NSCs (about 20 %) was obtained [31,51,52].

### 2.2 Treatments with single chemicals and mixtures and estimation of single chemical effective in vitro concentrations by Virtual Cell based Assay (VCBA)

After 7d of differentiation (7 DIV), NSCs were treated with single chemicals and their combined mixtures for 14d. The following chemicals were purchased: Bisphenol A (≥99 %, BPA), Chlorpyrifos (analytical standard, CPF), Lead(II) chloride (98 %, Lead), 2,2′4,4′-tetrabromodiphenyl ether (≥97 %, BDE47), Ethanol (≥99.8 % EtOH), Methylmercury(II) chloride (analytical standard, Methyl-Hg), PCB138 (analytical standard), Vinclozolin (analytical standard, Vincl) (all from Merck) (except for EtOH, these chemicals were dissolved in DMSO), 2,3,7,8-tetrachlorodibenzo-p-dioxin (≥98 %, TCDD) (Vogel GmbH, dissolved in DMSO), and Valproic acid sodium salt (≥98 %, VA) (Merck, dissolved in milliQ purified water) (Table 1). Assessment of cell viability using CellTiter-Blue® upon single treatment with BPA, CPF, Lead, Methyl-Hg, PCB138 and VA for 14d was already investigated in our previous study [31]. Table 1 shows the nominal concentrations used to assess cytotoxicity upon single treatment with BDE47, EtOH, Vincl and TCDD for 14d. Medium containing chemicals was refreshed twice/week.

**Table 1:** Nominal concentrations of BDE47, EtOH, Vincl and TCDD tested *in vitro* for the assessment of cell viability (14d treatment).

Chemical	Chemical class	Nominal concentrations tested in vitro	Range of concentrations found is human samples(Supplementary Table S1)
2,2′4,4′-tetrabromodiphenyl ether (BDE47)	Persistent organic pollutant	100, 25, 6.25, 1.56, 0.39, 0.10, 0.02 μM	6.1 × 10^−5^ - 0.038 μM
Ethanol (EtOH)	Industrial chemical	200, 50, 12.50, 3.13, 0.78, 0.20, 0.05 mM	6.9–102 mM
Vinclozolin (Vincl)	Fungicide	1340, 1000, 250, 62.5, 15.6, 3.9, 1.0 μM	0.00171−0.0065 μM
2,3,7,8-Tetrachlorodibenzo-p-dioxin (TCDD)	Persistent organic pollutant	830, 207.5, 51.88, 12.97, 3.24, 0.81, 0.2 nM	9.9 × 10^−4^ - 0.03 nM

Effective *in vitro* concentrations in Molar were estimated by using the Virtual Cell Based Assay (VCBA) to simulate *in vitro* fate, as described in [[53], [54], [55]] and as reported in ‘Supplementary Materials and Methods (M&M)_VCBA’. These results showed that between 85%–96% of chemicals were available to the cells (either as free concentration in medium, or sequestered by cellular lipids) (Table SM4, in Supplementary M&M_VCBA).

Following the approach described in our previous study [31], dose-response curves for cell viability were generated to identify, for each chemical, very low cytotoxic (IC_5_, concentrations causing a 5% decrease of cell viability), moderately cytotoxic (IC_20_, concentrations causing a 20 % decrease of cell viability) and non-cytotoxic (i.e., IC_20_ divided by 100, IC_20_/100) concentrations, compared with solvent control cultures (0.1 % DMSO). These three concentrations were studied to assess the effects of 14d treatments firstly with single chemicals on BDNF protein levels, neurite outgrowth and synaptogenesis, analysed by quantitative immunocytochemistry and high content imaging (HCI) analysis (Cellomics, ThermoFisher).

The HCI analyses enabled defining, for each chemical, the Lowest Observable Adverse Effect Concentration (LOAEC) (i.e., the lowest concentration where there is a statistically significant adverse effect) specific for each DNT endpoint (i.e., LOAEC-bdnf, LOAEC-neu, and LOAEC-syn), as summarized in Table 2. Raw prediction data in molar (M) of nominal concentrations indicated in Table 2 and chemical partitioning are reported in Table SM5 (Supplementary M&M_VCBA).

**Table 2:** **LOAECs specific for each DNT endpoint and their serial dilutions.** These nominal concentrations (in μM) were used to prepare the three mixtures described in Materials and Methods.

	BPA	CPF	Lead	BDE-47	EtOH	Methyl-Hg	PCB-138	VA	Vincl	TCDD	(μM)
**BDNF**	12.74	37.10	1.46	0.20	106,810	0.13	3.53	210	0.42	0.60	**LOAEC-bdnf**
	6.37	18.55	0.73	0.10	53,405	0.07	1.77	105	0.21	0.30	**LOAEC/2-bdnf**
	3.19	9.28	0.37	0.05	26,703	0.03	0.88	52.5	0.11	0.15	**LOAEC/4-bdnf**
	1.59	4.64	0.18	0.02	13,351	0.017	0.44	26.25	0.05	0.08	**LOAEC/8-bdnf**
**Neurite Outgrowth**	12.74	37.1	1.46	0.20	170,000	0.13	11.86	420	5.31	0.04	**LOAEC-neu**
	6.37	18.55	0.73	0.10	85,000	0.07	5.93	210	2.66	0.02	**LOAEC/2-neu**
	3.19	9.28	0.37	0.05	42,500	0.03	2.97	105	1.33	0.01	**LOAEC/4-neu**
	1.59	4.64	0.18	0.02	21,250	0.016	1.48	52.5	0.66	0.005	**LOAEC/8-neu**
**Synaptogenesis**	12.74	21.01	0.0073	17.62	106,810	0.05	0.06	2.1	0.42	0.60	**LOAEC-syn**
	6.37	10.51	0.0037	8.81	53,405	0.025	0.03	1.05	0.21	0.30	**LOAEC/2-syn**
	3.19	5.25	0.0018	4.41	26,703	0.013	0.015	0.53	0.11	0.15	**LOAEC/4-syn**
	1.59	2.63	0.0009	2.20	13,351	0.006	0.0074	0.26	0.05	0.08	**LOAEC/8-syn**

These LOAECs were calculated based on analysis of statistical significance (as detailed below), and were used to assess the effects of mixtures vs individual chemicals on cell viability and the selected DNT endpoints. To this end, the following three mixtures were created:

(i) a mixture combining five similar MoA chemicals (5-Sim): BPA, CPF, Lead, BDE47 and EtOH;
(ii) a mixture with five dissimilar MoA chemicals (5-Diss): Methyl-Hg, PCB138, VA, Vincl and TCDD; and
(iii) a mixture containing all 10 chemicals (10-All).

After 7 DIV, cells were treated for 14d with individual chemicals and their combined mixtures at the predefined LOAEC-bdnf, LOAEC-neu and LOAEC-syn concentrations and additional 2-fold dilutions. Mixture combined effects were compared with individual chemical effects, normalizing viability and HCI data to solvent control culture at the respective time point (14d treatment, corresponding to 21d of differentiation).

### 2.3 Analysis of cell viability with CellTiter-Blue®

After 7 DIV, cells were treated for 14d with single chemicals at the nominal concentrations indicated in Table 1, to determine cell viability and define concentrations corresponding to IC_20_/100, IC_5_ and IC_20_, compared with solvent control cultures (0.1 % DMSO). No significant differences between the solvent control (0.1 % DMSO) and medium control cells could be observed (data not shown). Analysis of cell viability was also performed to determine possible cytotoxic effects elicited by mixtures after 14d. Cell viability was measured by incubating cells with CellTiter-Blue® Reagent (final 1:6 dilution in cell culture medium) at 37 °C and 5% CO_2_ for 3−4 hours. After the incubation, 100 μL medium/reagent were transferred into new plates accounting also for wells containing blanc solution (medium with CellTiter Blue reagent), and fluorescence was measured at 530–560 nm-/590 nm (excitation/emission) in a multiwell fluorimetric reader (Tecan). After blanc subtraction, data were normalised to the mean of solvent control cells (0.1 % DMSO). IC_20_ and IC_5_ concentrations were calculated in GraphPad Prism 5 software using the Variable slope model to generate four-parameter dose-response curves.

### 2.4 Quantitative Immunocytochemistry (IC) and high content imaging (HCI) analysis

After 14d treatment, cells were fixed with 4% formaldehyde, washed twice with PBS 1X (w/o calcium and magnesium), and stored in PBS 1X at 4 °C prior to use. Cells were permeabilised in PBS 1X containing 0.1 % Triton-X-100 and 3.5 % bovine serum albumin (BSA) for 15 min at room temperature, and further incubated for additional 15 min with 3.5 % BSA in 1X PBS (blocking solution) to prevent nonspecific antibody binding. For the analysis of synaptogenesis, cells were stained with microtubule-associated protein-2 (MAP2, chicken, 1:3000, Abcam), synaptophysin (pre-synaptic marker) (SYP, rabbit, 1:300, Abcam), and post-synaptic density protein 95 (PSD95, mouse, 1:300, Abcam) specific antibodies, following a Thermo-Fisher standardised protocol (https://www.thermofisher.com/it/en/home/life-science/cell-analysis/cellular-imaging/high-content-screening/hcs-applications/hcs-synaptogenesis-assay.html). Analysis of neurite outgrowth was done by staining cells with an antibody specific for β-III-tubulin (mouse, 1:500, Thermofisher), along with an antibody specific for BDNF (rabbit, 1:70, Thermofisher) to measure BDNF protein at both cell body and neurite level, and glial fibrillary acidic protein (GFAP, chicken, 1:500, Abcam) to quantify the percentage of astrocytes. Primary antibodies were diluted in blocking solution and incubated overnight at 4 °C. Cells were washed twice with PBS 1X and further incubated for 45 min with fluorochrome-conjugated secondary antibodies (1:500, all from Abcam), and nuclei were counterstained with DAPI (1 μg/mL, Thermofisher). Quantification of mean fluorescence intensity and of the relative percentages of cell types was performed using the ArrayScan algorithm 'Neuronal Profiling V4.1′ bioapplication, as already described [31,56]. Through this algorithm, a specific nuclear mask is designed around the DAPI stained nuclei, distinguishing between live and pyknotic/dead cells, a mask identifying cell bodies is designed around the cell type antibody/antigen staining (i.e., MAP2, β-III-tubulin or GFAP). Other specific masks are designed to identify the neurite compartment and neurite branch points, and the fluorescence intensity levels of SYP and PSD95 puncta, or BDNF protein. The ArrayScan™ XTI High Content Platform (Cellomics) was set to take a minimum of 12 pictures/well at 10x magnification, and 6–8 internal replicates for each condition were considered. For qualitative analysis, 20x and 40x magnification pictures were also taken.

### 2.5 Electrophysiological measurements using multi-well microelectrode array (MEA)

NSCs were passaged and plated onto sterile polyethylenimine (PEI)- and laminin-coated 24-well microelectrode array (MEA) plates (24-Well Plate Glass (24W300/30G-288) V.232) (5 × 10^4^ cells/well, each well containing 12 gold microelectrodes) and cultured in the presence of neuronal differentiation medium. After 7 DIV, cells were treated for 14d either with individual chemicals or mixtures at LOAEC-syn and LOAEC/2-syn concentrations. Spontaneous electrical activity was recorded for 5 min before adding chemicals or solvent control (0.1 % DMSO) on day 0 and after 3, 7, 10 and 14d treatment. Spike rate (number of spike/sec), burst count (considering a burst as a train of at least 5 spikes occurring within 100 millisec), and network burst count (the number of synchronized bursts among all 12 electrodes in a well) were analysed. Electrical activity was recorded using the Multi-well MEA-System (Multi Channel Systems MCS GmbH), considering a Sampling Rate of 20,000 Hz, a Low-Pass Filter Cutoff Frequency of 3500 Hz, and a High-Pass Filter Cutoff Frequency of 100 Hz. Three internal replicates per condition were included in each experiment; final data represent the average of four independent biological experiments.

### 2.6 Benchmark Dose Modelling to compare the effects of individual chemicals vs mixtures

Parametrical dose response analysis for BDE47, EtOH, Vincl and TCDD was applied to the observed perturbation of each DNT specific endpoint after exposure to single chemicals for 14d by using the BMDExpress.2 open access software (https://github.com/auerbachs/BMDExpress-2/wiki) (Supplementary Fig. 1), as already described in our previous study [31]. This approach allowed to retrieve the Benchmark Dose (BMD) associated with a 5% change of response (BMD_5_). The upper (BMDU) and the lower (BMDL) bounds were also calculated to estimate the uncertainty of the BMD_5_ and assess experimental variability (Supplementary Tables S3-S6). To evaluate the potency of the individual chemicals in the mixtures and possible synergistic effects, we calculated, for each DNT endpoint, the Benchmark Response (BMR) of single chemicals considering the nominal concentrations used in the mixtures, according to the best-fit model calculated in the parametric dose-response analysis. The single chemical BMR values were compared with the measured mixture effects (normalized to untreated control). Moreover, the concentration addition approach and the Toxic Unit (TU) model [57] were applied, considering, for five chemicals, the following formula:

TU = [chem1]/BMD_5(chem1)_ + [chem2]/BMD_5(chem2)_ + [chem3]/BMD_5(chem3)_ + [chem4]/BMD_5(chem4)_ + [chem5]/BMD_5(chem5)_.

**Fig. 1:**
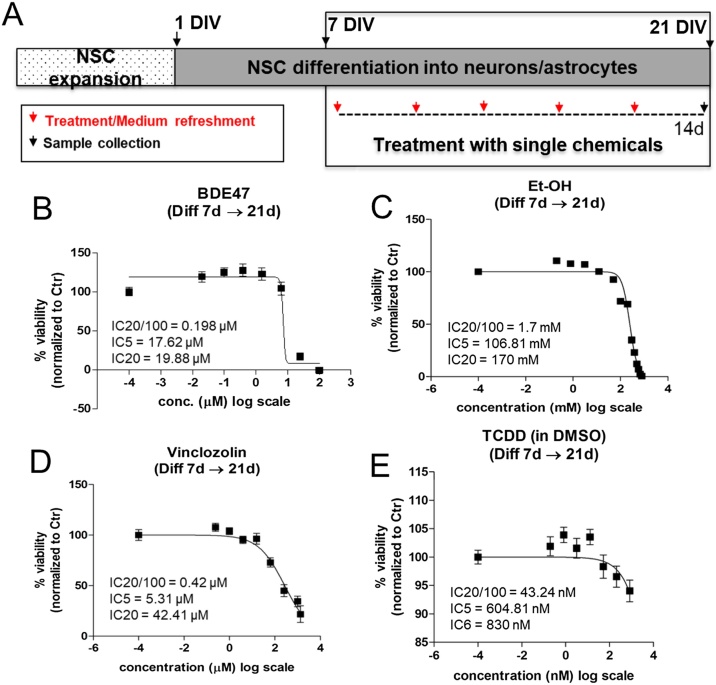
**Analysis of cell viability upon treatment with single chemicals in hiPSC-derived NSCs undergoing differentiation.** (A) NSCs were differentiated for 7 DIV and then treated for 14d with different nominal concentrations (as indicated) of: BDE47 (B), EtOH (C), Vincl (D) and TCDD (E), in comparison to solvent control (0.1 % DMSO, Ctr) at the respective time point. After 14d (i.e., 21 DIV), resazurin test was performed. All samples were normalised to solvent control (0.1 % DMSO, Ctr) at the respective time point. Chemicals were tested considering 6 internal replicates for each concentration (3-4 independent experimental replicates). Normalised values were imported into GraphPad Prism, where a non-linear fit (sigmoidal dose-response (variable slope)) was performed to calculate the inhibitory concentration (IC) values reported in (B-E).

A synergistic effect in mixtures was hypothesized when the following two criteria were both met:

(i) the TU calculated on the basis of single chemical contribution is ≤ 1 and the percentage of response experimentally observed in the mixtures is > 1 (i.e., a BMR > 5%); and

(ii) the observed mixture effects were at least (≥) two-fold higher in magnitude than the contribution of the most potent individual chemical.

When only the second criterion was met, an interactive mixture effect was hypothesized.

### 2.7 Correlation analysis to compare single chemical and mixture effects and the contribution of individual chemicals to the mixtures

Correlation plots and heatmaps were calculated according to Pearson to compare the effects elicited by individual chemicals and mixtures, and analyze the contribution of each chemical to overall mixture effects on each DNT endpoint. The correlation matrices were calculated considering the response across the concentrations corresponding to LOAEC/2, LOAEC/4 and LOAEC/8 specific for each DNT endpoint. Plots were generated using the Rcorrplot package.

### 2.8 Statistical analysis

Statistical significance of HCI and MEA data was assessed by one-way ANOVA with Dunnett's Multiple Comparison Test as Post Test, comparing all conditions vs solvent control (Ctr, 0.1 % DMSO) using the GraphPad Prism 5 software. All data represent the average of at least 3 biological replicates ± standard error mean (S.E.M.). For all graphs, an asterisk over a data point indicates a significant difference with the solvent control group at the respective time point (* p < 0.05, ** p < 0.01, *** p < 0.001).

## 3 Results

Human iPSC-derived NSCs were differentiated into a mixed culture of neurons and astrocytes for 21 DIV, as described in [31,51]. Previous characterization analyses of this hiPSC-derived NSC model (see e.g. [31,32]), showed that synaptogenesis, neurite outgrowth and neuronal and astrocyte differentiation started being upregulated after 7 DIV, increasing over time. Therefore, the effects elicited by a 14d repeated dose treatment with selected chemicals were explored in cells differentiated from 7d to 21d, to cover the early phases of synaptogenesis and neurite outgrowth, as alterations of any of these brain developmental processes can be particularly detrimental to the developing brain.

After 7 DIV, cells were treated for 14d with individually administered BPA, CPF, Lead, Methyl-Hg, PCB138, and VA, as described in [31], and with four previously untested chemicals (BDE47, EtOH, Vincl and TCDD) (Supplementary Table S1).

Concentrations considered for the analysis of selected DNT endpoints are relevant to human exposure based on concentrations found in human samples, as shown for the six previously tested chemicals [31,58] and the four new ones investigated in this study (see Table 1 and Supplementary Table S1). The nominal concentrations tested in this study are also in the range of concentrations described in previously published in vitro studies (e.g., [25,[59], [60], [61], [62]]). The effects of individually administered BDE47, EtOH, Vincl and TCDD are described in Results section 3.1, while effects triggered by three different types of mixtures (i.e., 5-Sim, 5-Diss, and 10-All) are reported in section 3.2.

### 3.1 Analysis of single chemicals effects

#### 3.1.1 Cell viability upon treatment with BDE47, EtOH, Vincl and TCDD

We previously analysed cell viability elicited by the single treatment with BPA, CPF, Lead, Methyl-Hg, PCB138, and VA for 14d [31]. Here, the same analysis was performed for individually administered BDE47, EtOH, Vincl, and TCDD (Fig. 1), previously not tested, in order to define non-cytotoxic (IC_20_/100), very low (IC_5_) and moderately cytotoxic (IC_20_) concentrations. Notably, a complete dose response curve for TCDD (Fig. 1E) could not be obtained due to the solubility limit of TCDD powder in DMSO (stock concentration = 830 μM) and, therefore, the highest tested concentration was 830 nM (corresponding to an IC_6_). Therefore, in all subsequent analyses we considered this concentration as the highest one for the assessment of TCDD effects on selected DNT endpoints.

#### 3.1.2 DNT endpoints: BDNF protein levels, neurite length and synapses

**Fig. 2:**
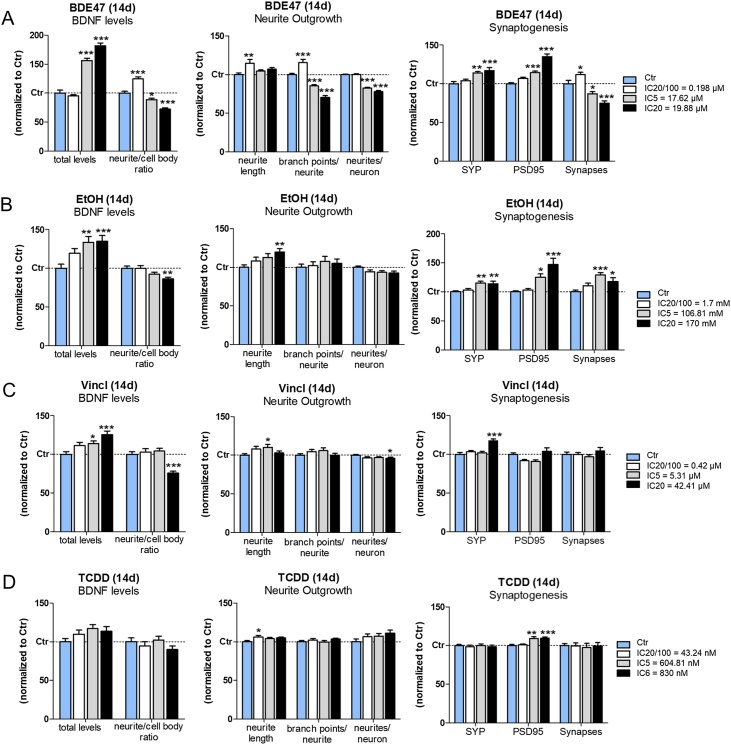
**DNT effects elicited by individually administered BDE47, EtOH, Vincl and TCDD.** NSCs were differentiated for 7 DIV and then treated for 14d with three different nominal concentrations (as indicated) of: BDE47 (A), EtOH (B), Vincl (C) and TCDD (D), in comparison to solvent control (0.1 % DMSO, Ctr, light blue bars) at the respective time point. Analysis of total BDNF levels and the neurite-to-cell body ratio of BDNF signal distribution (left), neurite outgrowth parameters (middle), and SYP, PSD95 and synapse numbers (right) was carried out, and data are represented as mean ± S.E.M. of 3-4 biological replicates.

The calculated IC_20_/100, IC_5_ and IC_20_ concentrations (and IC_6_ as the highest concentration for TCDD) for each chemical were considered to assess the effects of single chemical treatments for 14d on the following DNT endpoints (Fig. 2): BDNF protein levels (both total levels and the neurite-to-cell body ratio of BDNF levels observed in β-III-tubulin^+^ cells; neurite outgrowth measured by β-III-tubulin staining, considering neurite length, the number of branch points/neurite and the number of neurites/neuron; synaptogenesis (i.e., total level of both SYP and PSD95, and the number of synapses identified by the co-localization of both SYP^+^/PSD95^+^ puncta). Any statistically significant variation (i.e., an increase or a decrease) of the assessed neurodevelopmental features compared to solvent control at the respective time point, was considered as potentially indicative of DNT.

#### 3.1.3 Effects of 2,2′4,4′-tetrabromodiphenyl ether (BDE47)

An increase of total BDNF levels, along with a decrease of neurite-to-cell body ratio of BDNF protein level distribution at the highest tested concentration (IC_20_) compared to solvent control were observed after 14d treatment already at IC_5_ (50–70 % increase compared to solvent control) (Fig. 2A, see grey and black bars). Moreover, an increase of neurite length and number of branch points/neurite was observed at IC_20_/100 (Fig. 2A, white bars). Despite an increase of both SYP and PSD95 protein levels observed at IC_5_, the number of established synapses was found slightly decreased starting from IC_5_ concentration (Fig. 2A, grey bar).

#### 3.1.4 Effects of ethanol (EtOH)

A remarkable increase (by about 30 %) of BDNF total levels was found upon treatment with EtOH at IC_5_ concentration, along with a modest decrease of neurite to cell body ratio of BDNF protein level distribution at the highest tested concentration (IC_20_) compared to solvent control (Fig. 2B, grey and black bars). Additionally, a modest but significant increase in neurite length (by about 10 %) was visible at IC_20_ concentration, while the other neurite parameters did not significantly change (Fig. 2B).

A 10–15 % increase of both SYP and PSD95 protein level compared to solvent control was observed upon EtOH treatment already at IC_5_ (grey bars), which correlated with an increase in the number of synapses (SYP^+^/PSD95^+^ overlapping puncta) (Fig. 2B).

#### 3.1.5 Effects of Vinclozolin (Vincl)

The total levels of BDNF were found increased in a dose dependent manner (by 10–25 %), along with a significant decrease (by about 20 %) of BDNF neurite-to-cell body ratio observed at IC_20_ compared to solvent control (Fig. 2C).

A modest 10 % increase of neurite length was found upon treatment with Vincl at IC_5_ concentration, while the number of neurites/neuron was found slightly lower (by about 5%) at IC_20_ concentration (Fig. 2C).

No significant changes of synapse number was observed, while a modest increase (by about 15 %) of SYP levels was recorded at IC_20_ concentration (Fig. 2C).

#### 3.1.6 Effects of 2,3,7,8-Tetrachlorodibenzo-p-dioxin (TCDD)

With regards to total BDNF levels and neurite length, neither endpoints resulted significantly affected by TCDD treatment, except for a very modest upregulation of neurite length observed at the lowest tested concentration (IC_20_/100) (Fig. 2D, white bar).

After 14d treatment, both SYP levels and synapse number were found unchanged compared to solvent control, whilst a slight upregulation of PSD95 (by 10–15 %) was found at IC_5_ and IC_6_ concentrations (Fig. 2D, grey and black bars).

### 3.2 Analysis of chemical mixture effects

In a second step, the LOAECs specific for each individual chemical, for the three analysed DNT endpoints were calculated based on analysis of statistical significance. In this context, the LOAEC corresponds to the lowest tested concentration eliciting a statistically significant modification of at least one of the measured features. A comprehensive description of the effects induced by all 10 single chemicals, considering also the six chemicals previously tested in our study (i.e., BPA, CPF, Lead, Methyl-Hg, PCB138 and VA) [31], is reported in Supplementary Table S2.

Mixtures were prepared considering for each chemical the LOAEC eliciting a statistically significant modification of at least one of the measured DNT features. Concentrations were specific for each DNT endpoint and were therefore defined as LOAEC-bdnf, LOAEC-neu and LOAEC-syn. Three mixtures were created (i.e., 5-Sim, 5-Diss and 10-All) as described in Materials and Methods. Subsequently, three or four serial dilutions (by a factor of 2) of those mixtures were tested (Table 2). Raw prediction data in molar (M) of nominal concentrations shown in Table 2 and chemical partitioning are reported in Table SM5 in Supplementary M&M_VCBA. VCBA analysis showed that 85–96 % of chemicals were available to the cells either as free concentration in medium, or sequestered by cellular lipids (Table SM4 in Supplementary M&M_VCBA).

### 3.3 Cell viability

**Fig. 3:**
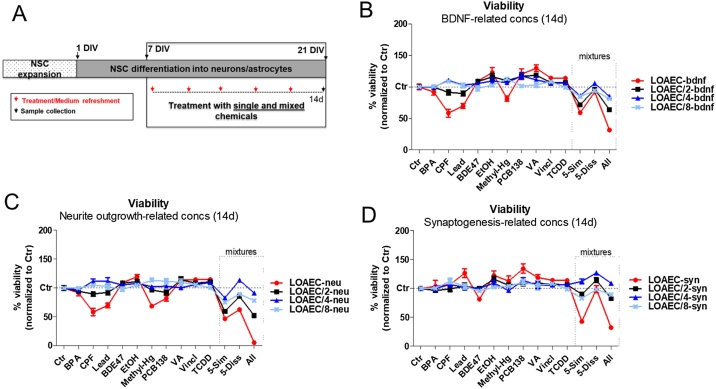
**Mixture effects on viability.** Analysis of cell viability upon treatments with mixtures in comparison with individual chemicals, using CellTiter-Blue assay. (A) NSCs were differentiated for 7 DIV and treated with individual chemicals or three different types of mixtures for 14d at different LOAECs specific for each DNT endpoint (as indicated in Table 2): BDNF (B), neurite outgrowth (C), and synaptogenesis (D). After 14d, resazurin test with CellTiter Blue was performed. All samples were normalised to solvent control medium (0.1 % DMSO, Ctr) at the respective time point. LOAECs (red curves) and their serial dilutions (respectively black (LOAEC/2), blue (LOAEC/4) and light blue (LOAEC/8) curves) were tested to assess whether mixed chemicals elicited cytotoxic effects. Mixture labelled as '5-Sim' contained similar MoA chemicals (affecting BDNF levels i.e., BPA, CPF, Lead, BDE47 and EtOH), whilst mixture with dissimilar MoA chemicals (i.e., Methyl-Hg, PCB138, VA, Vincl and TCDD) is labelled as '5-Diss'. The '10-All' mixture comprised all 10 chemicals together. Data are represented as mean ± S.E.M. of 3-4 biological replicates.

Cell viability analysis was performed after 14d treatment to evaluate possible cytotoxic effects elicited by mixtures in comparison with single chemicals (Fig. 3A). In general, some cytotoxic effects could be observed upon 14d treatment with 5-Sim mixtures at all tested LOAECs (-bdnf, -neu and -syn) (about 40–60 % decrease of viability compared to solvent control at the respective time point), and even more noticeable cytotoxic effects were found upon treatment with the mixtures containing all 10 chemicals at LOAEC concentrations (Fig. 3B-D, red curves), especially upon treatment with LOAEC-neu (Fig. 3C, red curve).

These cell viability analyses indicated that the 10 chemical mixtures were generally more cytotoxic than the two mixtures comprising only 5 chemicals, and that, between the 5-Sim and 5-Diss chemical mixtures, the one accounting for similar MoA chemicals (5-Sim) reduced cell viability more potently. In light of the high cytotoxic effects observed in particular upon treatment with ‘LOAEC-neu’ mixtures (red curve, Fig. 3C), LOAEC-neu mixtures were not considered in subsequent analyses, which aimed at assessing the effects of mixtures on the following DNT endpoints: BDNF protein levels, neurite outgrowth, synaptogenesis, the relative proportions of neurons and astrocytes (complemented by mathematical modelling), and the generation of spontaneous electrical activity, as described below.

### 3.4 Experimental and mathematical modelling analyses of DNT specific endpoints

#### 3.4.1 BDNF protein levels

**Fig. 4:**
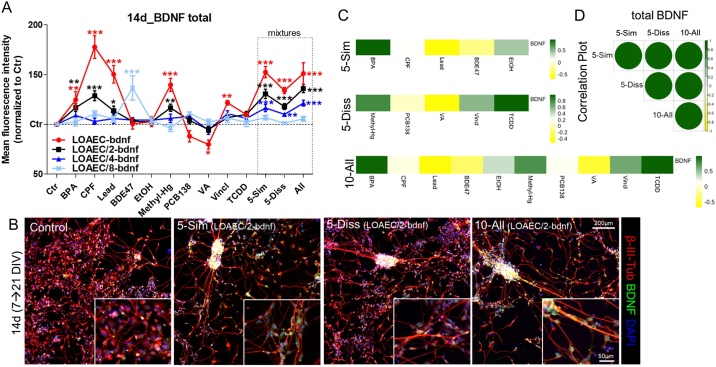
**Mixture effects on BDNF protein levels**. NSCs were differentiated for 7 DIV, and then treated for 14d with single chemicals or three types of mixtures as described in Fig. 3 legend. (A) Graph shows BDNF total levels measured upon treatment with single chemicals or mixtures at all tested LOAEC-bdnf concentrations (see Table 2). (B) Representative immunocytochemical image (at 10x magnification, with 40x magnifications insets) of cells treated with BDNF-related mixtures (at LOAEC/2-bdnf concentrations) for 14d and stained for β-III-Tubulin (red) and BDFN (green). For the analyses, all samples were normalised to solvent control (0.1 % DMSO, Ctr) at the respective time point. Data are represented as mean ± S.E.M. of 3-4 biological replicates. (C) Heatmaps showing the contribution of individual chemicals to overall mixtures’ effects (i.e., dark green: high correlation, same effect directionality; dark yellow: low correlation, opposite effect directionality). (D) Correlation plot to compare mixture effects (size and colour of the circles corresponds to the level of correlation between pair-wise mixtures: i.e., the bigger and darker green, the higher the level of correlation). C and D were calculated according to Pearson and the correlation matrix was calculated considering the response upon exposure to LOAEC/2-, LOAEC/4- and LOAEC/8-bdnf and effect trend/directionality.

After 14d treatment, majority of single chemicals induced a significant upregulation of BDNF levels (BPA, CPF, Lead, Methyl-Hg and Vincl) especially at the highest tested concentration (LOAEC-bdnf, red curve, Fig. 4A). Interestingly, BDE47 elicited a significant increase of BDNF levels only at the lowest tested concentration (LOAEC/8-bdnf, light blue curve, Fig. 4A), and VA caused a decrease of BDNF at the highest tested concentration. When combined in mixtures, both 5-Sim and 10-All did not elicit any potentiated/combined effects, since the observed concentration-dependent increase of BDNF levels were not greater than the effects elicited by the individual chemicals. Similarly, 5-Diss mixture increased BDNF levels in a concentration-dependent manner, although at a lower extent compared to the other tested mixtures (Fig. 4A, B); this increase was comparable to the effects observed upon treatment with Methyl-Hg alone, which suggests that Methyl-Hg may be the main driver of 5-Diss mixture effects on BDNF.

By looking at the correlation matrices summing up the contribution of individual chemicals to mixtures’ effects, BPA resulted as the major driver of BDNF upregulation in the 5-Sim mixture (Fig. 4C, dark green), while BDE47 and Lead were the chemicals contributing the least and driving opposite effects (Fig. 4C, yellow). Considering the 5-Diss mixture, the major drivers of BDNF protein upregulation were TCDD followed by Methyl-Hg, while the chemicals contributing the least were PCB138 and VA. In the 10-All mixture, BPA and TCDD, followed by Methyl-Hg and Vincl were the chemicals mainly contributing to overall mixture effects (Fig. 4C).

As indicated by correlation plot analysis, the three tested mixtures behave similarly one to each other with respect to BDNF level effects (Fig. 4D).

As described in our previous study [31], the Benchmark Response (BMR) of single chemicals were calculated for each DNT endpoint considering the nominal concentrations used in the mixtures in order to assess the potency of individual chemicals and possible synergistic effects in mixtures. A synergistic effect in mixtures was hypothesized when the following two criteria were both met:

(i) the TU calculated on the basis of single chemical contribution is ≤ 1 and the percentage of response experimentally observed in the mixtures is > 1 (i.e., a BMR > 5%); and

(ii) the observed mixture effects were at least (≥) two-fold higher in magnitude than the contribution of the most potent individual chemical.

When only the second criterion was met, an interactive mixture effect was hypothesized.

Looking at the BDNF level, neither of these two criteria was met (Supplementary Fig. 2); therefore, despite the increase in BDNF levels observed experimentally, no synergistic or interactive effects could be concluded through mathematical modelling.

#### 3.4.2 Neurite outgrowth

We explored the effects of single chemicals and mixtures also on neurite outogrowth and the percentages of neurons and astrocytes. To this end, cells were treated for 14d with nominal concentrations relevant to neurite outgrowth analysis (Table 2).

**Fig. 5:**
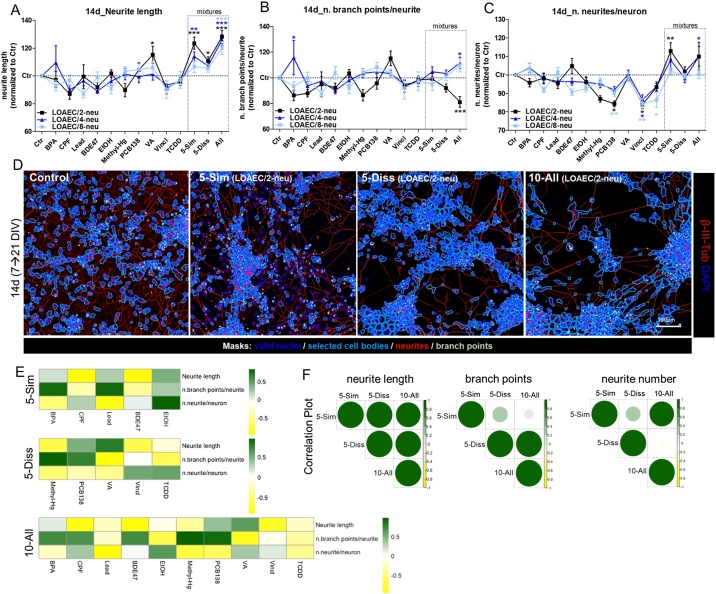
**Mixture effects on neurite outgrowth.** NSCs were differentiated for 7 DIV, and then treated for 14d with single chemicals and three types of mixtures as described in Fig. 3 legend. (A-C) Graphs report neurite length (A), the number of branch points/neurite (B), and the number of neurites/neuron (C) analysed upon 14d treatment with tested LOAEC-neu concentrations (see Table 2). (D) Representative immunocytochemical images (at 10x magnification) of cells treated with neurite outgrowth-related mixtures (at LOAEC/2-neu concentrations) and stained for β-III-Tubulin (red), along with masks to quantify valid nuclei (dark blue mask), selected neuronal cell bodies (light blue mask), neurites (red mask) and branch points (light green mask) by means of ArrayScan™ XTI High Content Platform (Cellomics). All samples were normalised to medium containing solvent only (0.1 % DMSO, Ctr). Data are represented as mean ± S.E.M. of 3-4 biological replicates. (E) Heatmaps comparing the effects elicited by individual chemicals and mixtures, and showing the contribution of each chemical to overall mixture effect (i.e., dark green: high correlation, same effect directionality; dark yellow: low correlation, opposite effect directionality). (F) Correlation plots to compare mixture effects (size and colour of the circles corresponds to the level of correlation between pair-wise mixtures: i.e., the bigger and darker green, the higher the level of correlation). E and F were calculated according to Pearson and the correlation matrices were calculated considering the response upon exposure to LOAEC/2-, LOAEC/4- and LOAEC/8-neu and effect trend/directionality.

While almost all analysed single chemicals caused only modest fluctuations over control levels of neurite-related parameters (Fig. 5A-C), VA at LOAEC/2-neu concentration was found to increase the length of neurites (by 15–18 %) (Fig. 5A, black curve). Converesely, PCB138, Vincl and TCDD generally decreased the number of neurites/neuron (by 12–15 %), and such effects were already visible at the lowest tested concentation (LOAEC/8-neu) (Fig. 5C, light blue curve).

Both the 5-Sim and the 10-All mixtures induced a significant dose dependent increase of neurite length (by about 23 % with 5-Sim, and by about 27 % with 10-All, at LOAEC/2-neu), with 5-Diss mixture causing less prominent effects (neurite length increased by about 10 % compared to solvent control at LOAEC/2-neu) (Fig. 5A, D). A similar trend was observed for the number of neurites/neuron, which increased in a concentration-dependent manner (by about 9–12 %) in cells treated with 5-Sim and 10-All mixtures, whilst 5-Diss mixture did not cause such changes (Fig. 5C, D). On the other hand, the number of branch points/neurite was found decreased (by about 18 %) by 10-All mixture at the highest tested concentration (LOAEC/2-neu) (Fig. 5B, D, black curve), and slighlty incresed at lower concentrations (LOAEC/4-neu and LOAEC/8-neu) (Fig. 5B, dark and light blue curves). Both 5-Sim and 5-Diss mixtures did not cause significant changes of branch point numbers at any tested nominal concentrations (Fig. 5B). Altogether, these data suggest induction of combined effects upon exposure to mixtures containing similar MoA chemicals (i.e., 5-Sim and 10-All) especially at lower concentrations (e.g., increase of neurite length upon treatment with 5-Sim and 10-All mixtures at LOAEC/4-neu, Supplementary Fig. 3A).

Correlation plot analysis showed that with regards to 5-Sim mixture, both CPF and BDE47 contributed in a counteractive manner to overall mixture effects on neurite length (Fig. 5E, yellow). Although the number of branch points/neurite was not significantly affected by the 5-Sim mixture (at all tested concentrations, see Fig. 5B), the chemicals behaving more closely to 5-Sim mixture were BPA and Lead (Fig. 5E, dark green). By looking at the number of neurites per neuron upon 5-Sim mixture exposure, EtOH resulted as the chemical contributing the most to this mixture effect, whilst BPA and Lead induced opposite effects (Fig. 5E).

Considering the 5-Diss mixture, the major driver of neurite length increase was VA (Fig. 5E), and with regards to the number of branch points per neurite, the chemicals behaving most similarly to 5-Diss mixture were Methyl-Hg and PCB138 (Fig. 5E).

Concerning the 10-All mixture, again VA was the main driver of neurite length increase induced by this mixture (Fig. 5E); on the other hand, branch points number was most potently affected by Methyl-Hg and PCB138, followed by BPA, CPF and BDE47 (Fig. 5E). Notably, EtOH was the chemical contributing the most to the 10-All mixture effects on neurite number (Fig. 5E).

Comparing the different tested mixtures in correlation plot analysis, the neurite feature that was affected in a comparable way both in terms of magnitude and effect directionality was neurite length (large dark green circles, Fig. 5F). While branch point number was similarly impacted by 5-Diss and 10-All, the number of neurites were comparably affected by the 5-Sim and 10-All mixtures (Fig. 5F).

Mathematical modelling showed that, although for these endpoints the evaluation of the contribution of single chemicals revealed a response above the 5% threshold (TU > 1), an interactive mixture effect could be hypothesized for neurite length analysed upon treatment with 10-All mixture at LOAEC/8-neu concentrations (indicated by the black dashed arrow in Supplementary Fig. 4). Indeed, for this specific endpoint and treatment, the response elicited by the mixture was at least two-folds of magnitude higher than the contribution of the most potent individual chemical.

#### 3.4.3 Percentages of neurons and astrocytes

**Fig. 6:**
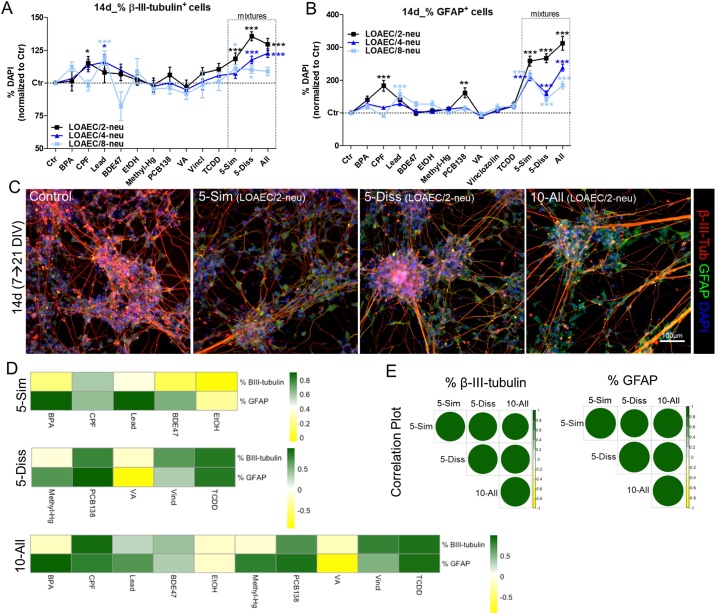
**Mixture effects on percentages of neurons and astrocytes.** NSCs were differentiated for 7 DIV, and then treated for 14d with single chemicals and three types of mixtures as described in Fig. 3 legend. (A, B) Graphs report percentage of β-III-tubulin^+^ (A) and GFAP^+^ cells (B) analysed upon 14d treatment with LOAEC-neu concentrations (see Table 2). (C) Representative immunocytochemical images (at 10x magnification) of cells treated with neurite outgrowth-related mixtures (at LOAEC/2-neu concentrations) and stained for β-III-tubulin (red) and GFAP (green). All samples were normalised to medium containing solvent only (0.1 % DMSO, Ctr). Data are represented as mean ± S.E.M. of 3-4 biological replicates. (D) Heatmaps comparing the effects elicited by individual chemicals and mixtures, and showing the contribution of each chemical to overall mixture effect (i.e., dark green: high correlation, same effect directionality; dark yellow: low correlation, opposite effect directionality). (E) Correlation plots comparing mixture effects (size and colour of the circles corresponds to the level of correlation between pair-wise mixtures: i.e., the bigger and darker green, the higher the level of correlation). D and E were calculated according to Pearson and the correlation matrices were calculated considering the response upon exposure to LOAEC/2-, LOAEC/4- and LOAEC/8-neu and effect trend/directionality.

We also assessed the percentage of neurons and astrocytes upon 14d treatment with single chemicals and mixtures. With regard to neuronal cell percentage, only CPF (at LOAEC/2-neu, black curve) and Lead (at the lowest tested concentrations, LOAEC/8-neu, light blue curve) caused an increase of β-III-tubulin^+^ neurons (by about 12–15 % compared to solvent control) (Fig. 6A). The other chemicals had no significant effects on the proportion of neurons. The percentage of astrocytes (identified by means of GFAP staining) was found remarkably increased upon treatment with CPF and PCB138 at LOAEC/2-neu (by about 90 % and 80 % respectively, compared to solvent control culture), and by Lead at LOAEC/8-neu (by about 50 %) (Fig. 6B), while the other chemicals did not impact significantly the percentage of GFAP^+^ cells.

Treatment with 5-Sim mixture increased the percentage of neurons at levels comparable or slightly higher that those found upon treatment with CPF only (about 15–20 % increase over control level, at LOAEC/2-neu) (Fig. 6A, C, black curve), while the percentage of astrocytes increased by almost 2.6 fold under the same treatment conditions (Fig. 6B, C, black curve). Significant increases of the GFAP^+^ cell percentage were found also with 5-Sim at lower tested concentrations (LOAEC/4-neu and LOAEC/8-neu) (Fig. 6B, dark and light blue curves).

Notably, a greater increase of both neuronal and astrocytic cell percentages was recorded after treatment with both 5-Diss and 10-All mixtures (neurons: at LOAEC/2-neu by about 30–35 % and 25–30 % respectively; astrocytes: at LOAEC/2-neu by about 2.7- and 3-fold respectively) (Fig. 6A-C, black curves), and such increase occurred in a concentration-dependent fashion. These results indicate induction of combined/potentiated effects induced by all tested mixtures at all tested concentrations (e.g., LOAEC/4-neu, Supplementary Fig. 3B). Among tested chemicals, CPF, Lead and PCB138 appeared as the major drivers of the neurotoxic effects in the mixtures where they were included (i.e., CPF and Lead were present in 5-Sim and 10-All, while PCB138 in 5-Diss and 10-All mixtures).

Looking at individual chemical contribution in the 5-Sim mixture, CPF was the chemical contributing the most to the increased percentage of β-III-tubulin^+^ neuronal cells (Fig. 6D). On the other hand, most of the chemicals present in the 5-Sim mixture (i.e., BPA, CPF, Lead and BDE47) contributed to the observed upregulation of GFAP^+^ cell percentage (Fig. 6D, green).

Concerning 5-Diss mixture, TCDD, PCB138 and Vincl mainly contributed to mixture effects on β-III-tubulin^+^ cell percentage increase, with also Methyl-Hg contributing to the upregulation of astrocyte percentage in this mixture (Fig. 6D). On the other hand, VA was the single chemical contributing the least to both these effects.

The effects induced by the 10-All mixture on neuronal cell percentage were more closely correlated to the effects induced by single treatment with CPF and TCDD, followed by PCB138 and Vincl (Fig. 6D). About GFAP^+^ cell percentage, most of the chemicals present in the 10-All mixture contributed to the overall mixture effects, with the exception of both EtOH and VA, showing counteracting effects compared to the mixture (Fig. 6D, yellow).

Correlation plot analysis showed that the three tested mixtures were highly correlated one to each other with respect to the effects induced on both neuronal and astrocytic cell percentages (Fig. 6E).

Mathematical modelling showed that only for the following parameters the response elicited by the mixture was at least two-fold higher in magnitude than the contribution of the most potent individual chemical: % neurons upon 5-Diss mixture at LOAEC/2-neu, and % astrocytes upon 5-Diss and 10-All mixtures at LOAEC/2-neu concentrations (Supplementary Fig. 5). Therefore, for these conditions, an interactive mixture effect was concluded.

#### 3.4.4 Synaptogenesis: SYP, PSD95 and number of synapses

**Fig. 7:**
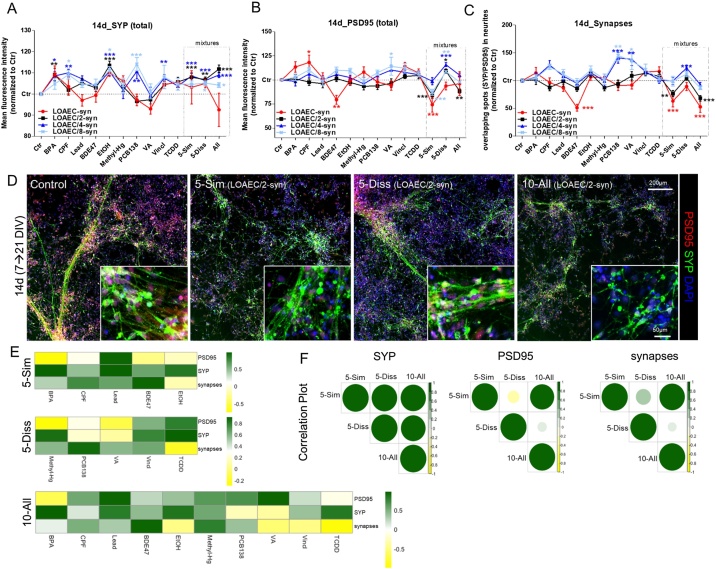
**Mixture effects on synaptogenesis.** NSCs were differentiated for 7 DIV and then treated for 14d with single chemicals and three types of mixtures as described in Fig. 3 legend. (A-C) Graphs report total levels of SYP (A), PSD95 (B), and number of overlapping SYP/PSD95 puncta (synapses, C) analysed upon 14d treatment with all tested LOAEC-syn concentrations (see Table 2). (D) Representative immunocytochemical image (at 10x magnification, with 40x magnifications insets) of cells treated with mixtures at different LOAEC/2-syn concentrations and stained for SYP (green) and PSD95 (red). All samples were normalised to solvent control (0.1 % DMSO, Ctr) at the respective time point. Data are represented as mean ± S.E.M. of 3-4 biological replicates. (E) Heatmaps comparing the effects elicited by individual chemicals and mixtures, and showing the contribution of each chemical to overall mixture effect (i.e., dark green: high correlation, same effect directionality; dark yellow: low correlation, opposite effect directionality). (F) Correlation plots comparing mixture effects (size and colour of the circles corresponds to the level of correlation between pair-wise mixtures: i.e., the bigger and darker green, the higher the level of correlation). E and F were calculated according to Pearson and the correlation matrices were calculated considering the response upon exposure to LOAEC/2-, LOAEC/4- and LOAEC/8-syn and effect trend/directionality.

After 14d treatment, upregulation of SYP (by 8–12 %) was observed in particular upon single treatment with BPA, CPF, EtOH, PCB138 and Vincl at lower concentrations (LOAEC/4-syn and LOAEC/8-syn) (Fig. 7A, dark and light blue curves). PCB138 and VA were the strongest inducers of synapse number increase (by about 40–47 %) at lower concentrations (LOAEC/4-syn and LOAEC/8-syn) (Fig. 7C, dark and light blue curves). On the other hand, treatment with BDE47 at the highest tested concentration (LOAEC-syn, corresponding to an IC_5_) caused a noticeable decrease of both PSD95 (by about 25 %) and the number of synapses (by about 50 %) (Fig. 7B, C, red curves).

Upon mixture treatments, the increase of SYP levels was not greater than the increase elicited by individual chemicals (about 8–12 % increase over control levels alredy at LOAEC/4-syn) (Fig. 7A, D), while a dose dependent decrease of both PSD95 (up to 25 % decrease vs control level at LOAEC-syn) and synapse number (up to 40 % decrease vs control, at LOAEC-syn) were observed in cells treated with 5-Sim mixture (Fig. 7B, C, red curves).

On the other hand, the mixture containing 5-Diss chemicals caused opposite effects, with an increase of both PSD95 and synapses (by about 20 %) observed at the lower concentrations (LOAEC/4-syn and LOAEC/8-syn) (Fig. 7B-D, dark blue and light blue curves).

The mixture with all 10 chemicals (10-All) was found to induce an increase of SYP (by about 8–10 %, already at LOAEC/8-syn), which anyway was not greater than the one induced by single chemicals (Fig. 7A, D), while levels of PSD95 did not change remarkably, except upon treatment with LOAEC/2-syn, which caused about 10 % decrease compared to control culture (Fig. 7B, D, black curve). Additionally, 10-All mixture caused a dose dependent decrease in the number of synapses (by 40–50 %), slightly greater than the effects induced by 5-Sim mixture (Fig. 7C, D). In conclusion, some combined/potentiated effects on synapse number decrease and PSD95 level reduction could be observed upon treatment with 5-Sim and 10-All mixtures at LOAEC/2-syn concentrations (as summarized in Supplementary Fig. 3C).

Overall these data indicate that chemical mixtures containing similar MoA chemicals (5-Sim) impaired synaptogenesis (i.e., increse of SYP, decrease of both PSD95 and the number of synapses) more prominently than the mixture containing dissimilar MoA chemicals (5-Diss), and the mixture with all 10 chemicals together caused similar or modestly greater effects than those observed upon treatment with 5-Sim mixture. Importantly, the chemical driving such neurotoxic effects in the mixtures was BDE47, especially when added at the highest tested concentration (LOAEC-syn, equal to IC_5_).

Correlation plot analysis showed that all chemicals present in 5-Sim mixture contributed to the increase of SYP levels, especially BPA, Lead and EtOH (Fig. 7E, dark green). The effects induced on PSD95 by single Lead treatment were closely correlated with those elicited by the 5-Sim mixture (Fig. 7E), whilst BDE47 was confirmed as the main driver of synapse number decrease induced by 5-Sim mixture (Fig. 7E).

Looking at 5-Diss mixture, Methyl-Hg and TCDD, followed by Vincl were the main drivers of mixture effects on SYP levels (Fig. 7E); TCDD and Vincl also resulted as the major inducers of 5-Diss mixture effects on PSD95 levels (Fig. 7E). PCB138 was the individual chemical that most closely correlated (and contributing to) to 5-Diss impact on synapse number (Fig. 7E).

Concerning the 10-All mixture, several chemicals contributed to the observed increase of SYP levels, with BPA, Lead, EtOH and TCDD as the main drivers of such effect (Fig. 7E, green and dark green). Moreover, Lead and VA were the major drivers of 10-All mixture effects on PSD95 levels (Fig. 7E, dark green). BDE47, followed by Methyl-Hg, resulted as the most potent inducers of 10-All mixture effects on synapse number decrease, whilst EtOH, VA, Vincl and TCDD inducing counteracting/opposite effects (Fig. 7E).

By comparing tested mixtures one to each other, the effects on SYP levels were highly correlated among all three mixtures (Fig. 7F); concerning PSD95 and synapse number, 5-Sim and 10-All resulted as most closely correlated, with 5-Diss behaving differently in comparison to both these two mixtures (Fig. 7F).

**Fig. 8:**
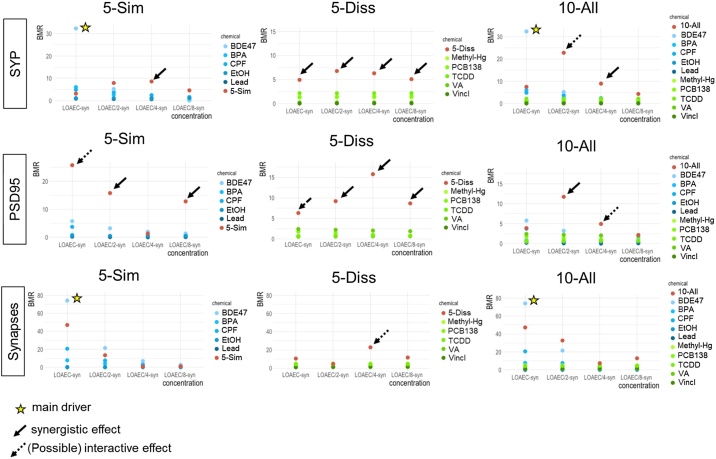
**Benchmark responses (BMR) for synaptogenesis.** The absolute BMR value of single chemicals, calculated considering their nominal concentrations used in the mixtures ('5-Sim', '5-Diss', and '10-All'), are plotted and compared with the absolute percentage of response observed experimentally in the mixtures for each DNT endpoint (normalised to control). For some of these synaptogenesis features/treatment conditions (indicated by solid black arrows), the contribution of single chemicals revealed a response below the 5% threshold (TU ≤ 1), the percentage of response experimentally observed in the mixtures was > 1 (i.e., BMR > 5%), and the response induced by the mixture was at least two-folds of magnitude higher than the contribution of the most potent individual chemical. For those synaptogenesis features/treatment conditions indicated by dashed black arrows an interactive mixture effect can be hypothesized when the response elicited by the mixture was at least two-fold higher in magnitude than the most potent individual chemical, although the evaluation of the contribution of single chemicals revealed a response above the 5% threshold (TU > 1). Stars next to a single chemical (BDE47) indicate that the chemical may be considered as the main driver of mixture effects.

Mathematical modelling indicated that for some synaptogenesis features/treatment conditions (e.g., SYP level upon treatment with 5-Diss mixture at all tested concentrations, PSD95 levels upon treatment with 5-Sim at LOAEC/2- and LOAEC/8-syn, and upon treatment with 5-Diss and LOAEC/2-, LOAEC/4- and LOAEC/8-syn, and all the other conditions indicated by the solid black arrows in Fig. 8), the contribution of single chemicals showed a response below the 5% threshold (TU ≤ 1), as well as a mixture response of at least two-folds of magnitude higher than the contribution of the most potent individual chemical. As for those synaptogenesis features and treatment conditions both selected criteria were met, a synergistic effect was concluded.

For other synaptogenesis features (i.e., SYP upon treatment with 10-All at LOAEC/2-syn; PSD95 upon treatment with both 5-Sim and 5-Diss at LOAEC-syn; PSD95 upon treatment with 10-All at LOAEC/4-syn; and synapses upon treatment with 5-Diss at LOAEC/4-syn, as indicated by dashed black arrows in Fig. 8), an interactive mixture effect could be hypothesized, considering that the response elicited by the mixture was still two-folds of magnitude higher than the contribution of the most potent individual chemical. BDE47 (present in both 5-Sim and 10-All mixtures) may be the main driver of mixture effects on SYP and on the number of synapses (upon treatment with both 5-Sim and 10-All at LOAEC-syn) (Fig. 8, indicated by a star).

#### 3.4.5 Electrical activity

**Fig. 9:**
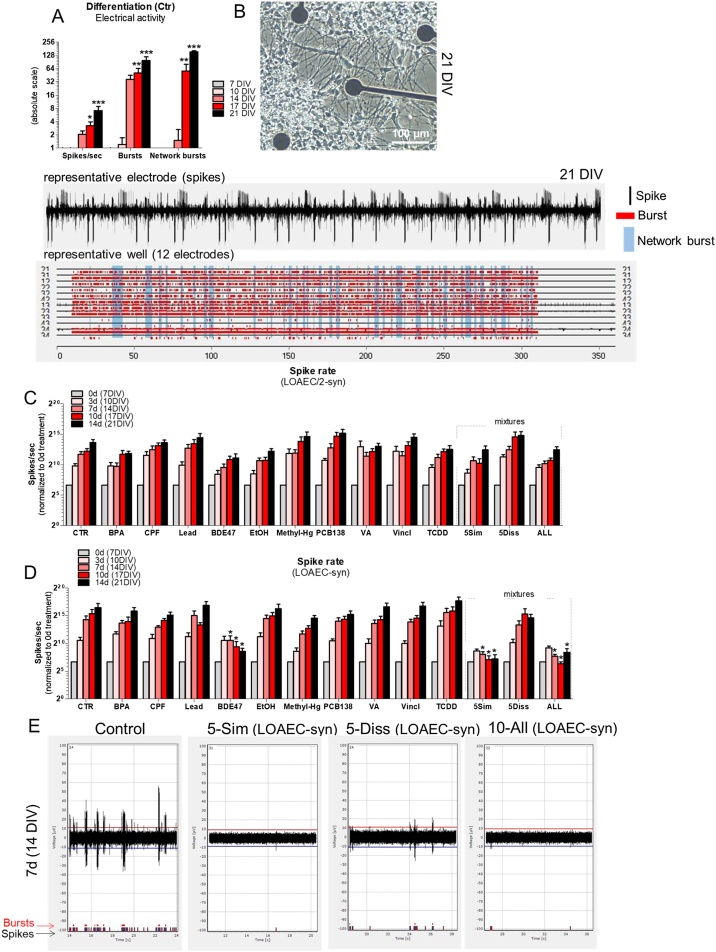
**Mixture effects on electrical activity.** (A) Graph shows spike rate (spikes/sec), number of bursts and number of network bursts after 5 min recording (raw data, absolute scale) analysed in cells differentiated for 21 DIV. Electrical activity was recorded after 7, 10, 14, 17 and 21 DIV. (B) Representative phase contrast image of NSC-derived neuronal cells cultured on a MEA for 21 DIV, along with representative raster plots (5 min (300 s) recording over 6 min total reading) of one single electrode and a set of 12 electrodes within a well. (C, D) Graphs report spike rate (number of spikes/sec) recorded after 0, 3, 7, 10 and 14d treatment with either single chemicals or mixtures at the concentration of LOAEC/2-syn (C) and LOAEC-syn (D) or solvent control (Ctr, 0.1 % DMSO) and normalised to 0d treatment. (E) Representative image of spikes and bursts occurring during about 10 s recording in cells after 7d treatment (14 DIV) with 5-Sim, 5-Diss and 10-All mixtures at LOAEC-syn, or solvent control (0.1 % DMSO). Data are represented as mean ± S.E.M. of 3-4 biological replicates.

Analysis of spontaneous electrical activity by MEA showed a progressive increase of spike rate (number of spikes/sec), the number of bursts and the number of synchronized bursts (network bursts) in the control culture undergoing neuronal differentiation for 21 DIV (Fig. 9A, B).

Notably, neuronal network formation was not significantly perturtbed upon exposure to single chemicals or mixtures at LOAEC/2-syn concentrations upon 0, 3, 7, 10 and 14d treatment (Fig. 9C). On the other hand, BDE47 at LOAEC-syn (corresponding to IC_5_) caused a decrease of both spike rate (Fig. 9D), and the number of bursts (Supplementary Fig. 6B) already after 7d treatment. Moreover, the number of synchronized bursts (or network bursts), indicative of a mature neuronal network, resulted significantly decreased upon 14d treatment with BDE47, CPF, EtOH and Methyl-Hg at LOAEC-syn (Supplementary Fig. 6C).

Upon treatment with both 5-Sim and 10-All mixtures, all measured parameters (spike rate, bursts and network bursts) resulted dramatically decreased starting from 7d treatment (Fig. 9D, E and Supplementary Fig. 6), while 5-Diss mixture did not cause significant effects compared to solvent control. These data indicate that under these treatment conditions, BDE47 was the major driver of decreased neuronal electrical activity in the two mixtures where it was included (5-Sim and 10-All). Other chemicals, i.e., CPF, EtOH and Methyl-Hg, after 14d caused a significant disruption of network bursts, suggesting delayed neurotoxic effects on neuronal network formation and function.

### 3.5 Comparison of different mixtures’ effects (3-Sim, 5-Sim, 3-Diss, 5-Diss, 6-All and 10-All)

**Fig. 10:**
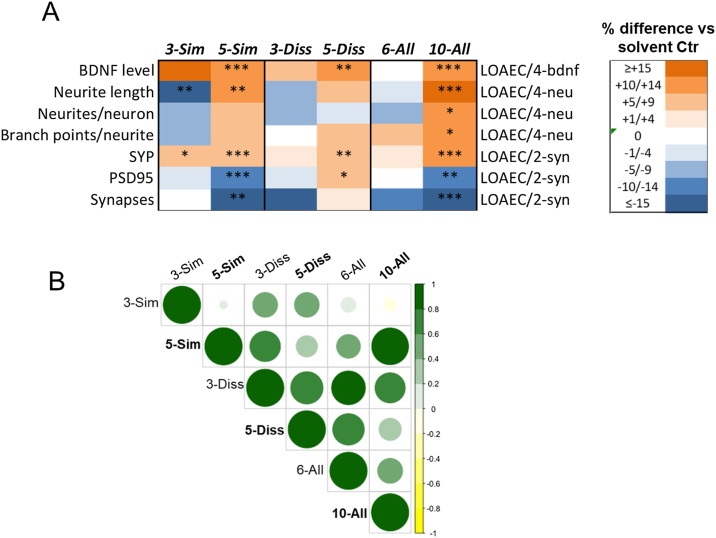
(A) Heatmap summarizing the effects of 14d treatment with mixtures tested in our previous study [31] (i.e., 3-Sim: BPA + CPF + Lead; 3-Diss: Methyl-Hg + PCB138+VA; 6-All: BPA + CPF + Lead + Methyl-Hg + PCB138+VA) in comparison with mixtures tested in this study (i.e., 5-Sim: BPA + CPF + Lead + BDE47+EtOH; 5-Diss: Methyl-Hg + PCB138+VA + Vincl + TCDD; 10-All: BPA + CPF + Lead + BDE47+EtOH + Methyl-Hg + PCB138+VA + Vincl + TCDD). Delta values (% difference vs solvent control) between indicated representative LOAECs are displayed (different colours refer to ranges of delta values, orange: increase; blue: decrease). Asterisks indicate statistical significance compared to solvent control (* p < 0.05, ** p < 0.01, *** p < 0.001). (B) Correlation plot summarizing the overall effects (i.e., Delta values, % difference vs control) considering mixtures’ effects on all the DNT endpoints at the concentrations shown in A. The correlation matrix was calculated using Pearson and visualized using Rcorrplot package. Size and colour of the circles corresponds to the level of correlation between pair-wise mixtures: i.e., the bigger and darker green, the higher the level of correlation.

Fig. 10 summarizes the main DNT effects experimentally observed upon 14d treatment with 5-Sim, 5-Diss and 10-All mixtures at the indicated representative (non-cytotoxic) LOAECs compared with the effects triggered by less complex mixtures (i.e., 3-Sim, 3-Diss and 6-All) tested in our previous study [31]. All tested mixtures generally induced an upregulation of BDNF protein levels (except for 6-All mixture, which caused no effects at this tested nominal concentration). Opposite effects on neurite-related features could be observed comparing the two studies, with less complex mixtures inducing a dowregulation and more complex mixtures inducing an upregulation of neurite outgrowth over solvent control levels. On the other hand, similar effects on synaptogenesis-related markers (SYP, PSD95 and number of synapses) were observed comparing ‘Sim’ and ‘All’ mixtures tested in the two studies, but such effects resulted greater upon treatment with more complex mixtures (in particular 5-Sim and 10-All, which caused a greater decrease of both PSD95 and synapses).

These data globally suggest that DNT effects do not always result greater by increasing the number of chemicals within a mixture, and that such effects may be diverse in both magnitude and direction depending on the analyzed DNT endpoint.

Finally, the overall level of correlation between mixtures is indicated in Fig. 10B, considering all the DNT endpoints and concentrations shown in Fig. 10A. This comparative analysis mainly indicated that:

(i) the overall effects induced by the 3-Sim mixture are very different (in magnitude) from the effects induced by the 5-Sim mixture; on the other hand, the overall effects induced by the 3-Diss mixture are quite similar (in both magnitude and direction) to the effects induced by the 5-Diss mixture;

(ii) the overall effects induced by the 5-Sim mixture are very similar (in both magnitude and direction) to the effects induced by the 10-All mixture;

(iii) on the other hand, the overall effects induced by the 3-Sim mixture are very different (both in magnitude and direction) from the effects induced by the 10-All mixture.

This suggests that the addition of EtOH and BDE47 had the highest impact on the overall effects induced by both 5-Sim and 10-All mixtures, and that these chemicals may most likely be the main drivers of the DNT effects observed upon treatment with those mixtures.

## 4 Discussion

A possible concern from combined exposure to multiple chemicals spanning several chemical classes has been demonstrated in several case studies (examples in [63]). In the context of the EDC-MixRisk project based on the SELMA mother-child cohort, Bergman et al. [64] detected 41 chemicals in pregnant women, of which several were associated with adverse health outcomes in neurodevelopment (i.e., language delay at 30 months of age, and cognitive functions at 7 years of age). Larsen et al. [65] looked into exposure and potential risks from endocrine disrupting chemicals and neurotoxicants in children under 3 years and pregnant women/unborn children. Neurotoxicants included substances such as BPA, dioxins, PCBs, acrylamide, methyl mercury, several of them also used in our current study. Performing a risk assessment based on concentration addition and exposure to these substances via food, dust and articles, and breast milk indicated exposure doses of concern in particular for children under 3 years. Both studies confirm that combined exposure is relevant across chemical classes considering effects on the same adverse outcome.

Exposure to chemical mixtures, which more closely reflect real-life exposure, induces DNT effects, perturbing BDNF levels, synaptogenesis and neuronal network formation and function, which have been identified as common KEs in several DNT-relevant AOPs [31,32], leading to impairment of learning and memory in children. Moreover, perturbation of these neurodevelopmental processes vital for normal brain development seems to be more prominent upon treatment with chemicals working through similar MoA (in this case, perturbation of BDNF expression and/or signalling), confirming our previous study [31].

As a follow-up of our previous study on BPA, CPF, Lead, Methyl-Hg, PCB138 and VA and their mixtures (with 3 or 6 chemicals) [31], in the present study we assessed the effects of four additional chemicals that were not previously tested: BDE47, EtOH, Vincl and TCDD, creating more complex mixtures with either 5 or 10 chemicals on the basis of their MoA. These chemicals were selected because of their proven association with the risk of neurodevelopmental deficits (Supplementary Table S1). In particular, prenatal and childhood exposure to BDE47 (included in the newly tested mixtures) and other PBDEs has been found associated with poorer attention, fine motor coordination and cognition in children [66]. Additionally, prenatal exposure to EtOH has been associated with multiple behavioural and cognitive attention deficits in rats, similar to the effects observed in foetal alcohol syndrome and attention deficit and hyperactivity disorder (ADHD) [[67], [68], [69]]. Vincl can cause severe and widespread neuroendocrine disruptions in specific brain regions (e.g., hippocampus, amygdala, hypothalamus), leading to behavioural changes (e.g., cognitive deficits) in different species [70], with alteration in the expression of genes encoding for components of excitatory glutamatergic synapses, migration and pathfinding control, glutamatergic and GABAergic neurons, and genes linked with increased risk of ASD, as shown in perinatally exposed rats at PND 6 [71], and alteration in the proportions of different neuronal subpopulations as observed in perinatally exposed Dutch-belted rabbit (*Oryctolagus cuniculus*) offspring [72]. On the other hand, TCDD has been linked to a decrease of composite motor and gross motor scores in boys [73].

We created mixtures with either 5 or 10 chemicals, considering their MoA, and our data indicate that mixtures of chemicals working through similar MoA at low concentrations tend to increase BDNF levels, neurite length and overall number of neurites/neuron, whilst decreasing synapse formation and neuronal electrical activity. Additionally, mixtures induced a prominent increase in the percentage of astrocytes, along with a more modest increase of neuronal cell percentage.

By comparing the effects of chemical mixtures tested in our previous study [31], opposite effects on neurite-related features could be observed comparing the two studies: less complex mixtures (with 3 or 6 chemicals) induced downregulation, while more complex mixtures (with 5 or 10 chemicals) induced upregulation of neurite features. On the other hand, with regard to synaptogenesis features, the impact of more complex mixtures was generally greater than the one induced by less complex mixture, as shown e.g., by the more prominent decrease of synapse number upon treatment with 5-Sim and 10-All (Fig. 10A).

Comparative analysis of overall mixture effects (considering all major DNT endpoints tested in the two studies) (Fig. 10B), further showed that 3-Sim mixture effects were globally very different from 5-Sim mixture effects, whilst 3-Diss and 5-Diss mixture effects did not differ. This suggests that adding two similar MoA chemicals (BDE47 and EtOH) to the mixture had greater DNT effects than adding two chemicals acting through dissimilar MoA (Vincl and TCDD).

Notably, all mixtures tested in our previous experiments [31] and the present study generally induced an upregulation of BDNF protein levels. Chemicals working through similar MoA target the same signalling pathway and may, therefore, more potently and eventually irreversibly compromise cellular defence mechanisms. Activation of BDNF signalling could contribute to increase neuronal and glial proliferation, since BDNF plays an important role in neuronal proliferation, survival and differentiation [74,75]. Additionally, astrocytes have been reported to express and release BDNF [76,77], indicating that astrocytes may regulate BDNF availability to neurons [78]. The observed increase of BDNF levels could stimulate astrocyte proliferation especially upon treatment with 5-Sim chemicals, confirming their neuronal protective role. It will be interesting to investigate whether the observed increase of BDNF level originate from neurons, astrocytes or both cell types.

Upregulation of BDNF levels may occur as a prosurvival mechanism; however, this may not be sufficient to prevent the neurotoxic effects observed e.g., on synapse formation (Fig. 10). Further studies should explore the dynamics of these DNT effects over time.

Depending on the DNT endpoint, different chemicals appeared to work as major drivers of the neurotoxic effects observed in mixtures. The neurotoxic effects induced by these chemicals in relation to the analysed DNT endpoints are discussed in the following sections.

### 4.1 Chemicals causing an increase of BDNF levels

With regard to BDNF levels, correlation plot analysis of individual chemical contribution to mixture effects indicates that BPA, TCDD and Methyl-Hg were the most plausible drivers of BDNF level increase upon treatment with mixtures. Notably, also CPF and Lead (at LOAEC-bdnf and LOAEC/2-bdnf) and BDE47 (at low concentration, LOAEC/8-bdnf) induced an upregulation of BDNF levels, although their contribution to mixture effects was low. On the contrary, VA counteracted this effect and was found to elicit a decrease of BDNF level at the highest tested concentration (LOAEC-bdnf).

Several studies have reported direct effects of tested chemicals on BDNF expression or protein levels (see Supplementary Table S1 and [31]). For instance, upregulation of BDNF expression was observed specifically in the brain of female rat offspring exposed to BPA during gestation, whilst a decrease of BDNF expression was observed in male rats offspring [79], which is in line with a previous study showing a decrease of CREB phosphorylation and BDNF in the hippocampi of male rats prenatally exposed to BPA; these animals also showed impairments in object recognition memory [50].

Furthermore, rats acutely exposed to Methyl-Hg via oral administration, were found to undergo an increase of BDNF levels, which was linked to alteration of gut-brain axis related metabolites [80]. Increased expression of BDNF was found in astrocytes of the inferior colliculus (a brain region involved in auditory control) in Methyl-Hg-exposed mice [81]. Increased BDNF levels were also found in the cerebellum of adult male mice exposed for 2 weeks to 40 μg/mL Methyl-Hg (diluted in drinking water, *ad libitum*), which also showed motor impairments characteristic of cerebellar toxicity [82]. Conversely, decreased BDNF protein levels was observed in the hippocampi of rat offspring prenatally exposed to Methyl-Hg [83]. Additionally, rat cortical neurons treated with 1 μM Methyl-Hg underwent decrease of BDNF both at gene and protein level [84].

With regard to CPF, an increase of CREB phosphorylation (and consequentially BDNF levels) has been reported in primary cortical and hippocampal neurons [85]. Increased BDNF expression was reported also in the brain of adult Zebrafish exposed to CPF [86]. Upregulation of BDNF expression was reported in the hippocampus of rats exposed to CPF at 10 mg/kg/d for 21d [22]. Upregulation of BDNF gene expression was also found in PC12 cells undergoing differentiation towards neurons, and exposed to 30 μM CPF for 24 or 72 h [25]. On the other hand, a decrease of BDNF levels in CA1 area of the hippocampus was observed in adult rats IP injected with CPF (3 mg/kg body weight, 5d a week, for 6 consecutive weeks) [87]. Analogously, decrease of cortical BDNF gene expression was observed in adult rats administered for 4 weeks with 10 mg/kg CPF [88].

Opposite to our data, rats prenatally exposed to Lead, showed a decrease of cerebellar BDNF levels on PND 21 [89]. Additionally, mice prenatally exposed to Lead showed an increase of pro-BDNF in the hippocampus, along with a decrease of mature BDNF, CREB, and phosphorylated CREB compared with the control group at the age of 4, 13 and 16 months [90]. Downregulation of BDNF gene expression was also observed in zebrafish embryos exposed to Lead (0, 5, 10, and 20 μg/L) starting from 6 h post-fertilization (hpf) until 144 hpf [91]. Epidemiological studies on Lead exposure found different effects, such as no association between maternal blood levels of Lead and BDNF in women in the first trimester of pregnancy [92], and an inverse association between blood levels of Lead and serum BDNF concentrations in male children (but not in girls) [93].

Moreover, the frontal lobes of offspring rats exposed to BDE47 during gestation, at postnatal day (PND) 41, showed a decrease of nuclear 5-methylcytosine in Bdnf gene, possibly indicative of higher BDNF expression [94].

It should be pointed out that any dysregulation (up or down) of BDNF expression and/or protein level may be indicative of DNT. Additionally, the selection of experimental model and conditions (e.g., in vivo vs in vitro, gestational vs adult age of animals, female vs male sex, acute vs repeated/chronic exposure times, selected concentrations, etc.) appear to be critical factors influencing the sensitivity of the experimental model to chemicals and their possible effects on BDNF signalling. Importantly, increased BDNF levels have been reported in epidemiological studies on children affected by autism spectrum disorder (ASD) [95,96], as further discussed in the following section (“*Potential associations between observed perturbations of measured DNT endpoints and neurodevelopmental deficits in children*”).

### 4.2 Chemicals perturbing neurite outgrowth and the proportion of neurons and astrocytes

A dose-dependent increase of both neurite length and the number of neurites/neuron was observed upon exposure to all mixtures, in particular 5-Sim and 10-All, with VA resulting as the strongest inducer of neurite length increase (Fig. 5E). In line with this, single treatment with VA has been reported to increase neurite length in human neuroblastoma cells [97], in mouse embryonic NSCs [98], in primary rat cortical neurons [99], in spiral ganglion NSCs [100], and in a murine Alzheimer's disease model [101]. Prenatal exposure to VA has been shown to increase the risk of ASD-related features, including neural tube developmental defects and imbalance of excitatory/inhibitory synapse [[102], [103], [104]].

Notably, compared to our previously published study on mixtures with 3 or 6 chemicals [31], which were found to shorten neurite length upon 14d treatment, these more complex mixtures with 5 or 10 chemicals (accounting also for BDE47 and EtOH (in 5-Sim), and Vincl and TCDD (in 5-Diss)) induced opposite effects with respect to this specific DNT endpoint (i.e., increase of neurite outgrowth) (see Fig. 10). It should be considered that any change (up- or down-regulation) of neurite-related features (i.e., neurite length, overall neurite numbers, number of branch points) over control levels should be considered as a possible DNT effect, as such alterations in neuronal differentiation may have an impact on neuronal network formation and function, as observed based on neuro-electrophysiological measurements in this study.

Furthermore, assessment of individual chemical contribution to mixture effects (Fig. 6D) indicates that BPA, CPF, Lead, Methyl-Hg, PCB138 and TCDD resulted as the major drivers of the observed increased percentage of neurons and, more prominently, astrocytes upon mixture treatments (i.e., BPA, CPF and Lead were present in 5-Sim; Methyl-Hg, PCB138 and TCDD were present in 5-Diss; all these chemicals were included in 10-All mixture).

Increase of GFAP expression and protein levels has been reported in C8-D1A mouse astrocyte cells treated with 30 μM BPA [105], in serum-free mouse embryo (SFME) cells treated with BPA up to 100 pg/mL (4.4 × 10^−7^ mM) [106], as well as in female juvenile mice upon exposure to BPA through maternal diet (50 mg/kg diet), which caused anxiety-like behaviour [107]. Similarly, increase of GFAP^+^ astrocytes has been observed in the cingulum of offspring rats prenatally exposed to BPA (1 mg/L of drinking water) [108], and an increase in astroglia density has been described in the medial prefrontal cortex and hippocampus of adult male rats treated with BPA (300 μg/kg body weight) [109]. Alterations of astrocyte proportion may affect functional and behavioural outputs. For instance, Nagai et al. found that activation of Gi-mediated GPCR signalling in striatal astrocytes in vivo was sufficient to alter mouse behaviour [110]. A more comprehensive review on this topic highlights the crucial role of astrocytes in the regulation of sleep, circadian rhythm, memory formation, mood-associated behaviours (including anxiety disorders), and nutritional homeostasis [111].

Two day treatment with CPF has been shown to inhibit neurite outgrowth in a dose-dependent manner in human PSC-derived neuronal cells, starting at 10 μM, but co-culture with astrocytes protected neurons from the effects of CPF at higher concentrations (including 30 μM) [112]. CPF has also been shown to decrease dopaminergic neuronal cell number and to increase microglia and astrocytes in the substantia nigra of newborn rats treated daily with CPF from PND11 to PND14 [113]. Similarly, GFAP immune-reactivity has been found increased in astrocytes of Wistar rats gavaged daily with CPF [114], in the forebrain of rats exposed to CPF from PND1 to PND6 [115], as well as in the piriform cortex, motor cortex and the basolateral amygdala [116], and in the hippocampus [117] of adult mice treated with CPF. Notably, prenatal and infant exposure to CPF has been associated with the risk of ASD in children [118]; decrease of GABAergic receptors, insufficient gestational thyroid hormones, oxidative stress, mitochondrial dysfunctions and induction of neuroinflammation are considered as some of the most plausible mechanisms underlying CPF effects and its association with ASD risk [119]. Reduced expression of nerve growth factor, reelin, the muscarinic acetylcholine receptor, and myelin-associated glycoprotein, as well as increased expression of GFAP indicative of astrocyte reactivity have been reported as possible mechanisms underlying CPF effects on the developing brain in an in vivo study in rats [115].

Lead overload has been associated with the risk of ASD [120] and it is known to induce both micro- and astro-gliosis in the brain, triggering the production of pro-inflammatory cytokines [121]. Chronic Lead exposure has been shown to increase the percentage of astrocytes in the spinal cord of rats [122], and to induce microgliosis and astrogliosis in the hippocampus of young mice via activation of TLR4-MyD88-NFκB signalling pathways [123]. An increase of GFAP^+^ astrocytes was also demonstrated in NSCs derived from the hippocampus of newborn and adult rats treated with 0−200 μM Lead [124]. Contrary to our results, Lead treatment for 7d (0.97 μM, a concentration slightly higher than the ones tested in our study, i.e., 0.18−0.73 μM) was found to decrease the number of both astrocytes and neurons in primary cultures of embryonic rat hippocampal cells [125]. Altogether, these studies point to a dual role played by astrocytes upon Lead exposure: under specific conditions (e.g., low toxic concentrations and/or short exposure times), they may play a neuroprotective role; however, if their buffering capacity is compromised due to e.g., exposure to highly toxic concentrations and/or prolonged/chronic exposure, they may contribute to neuronal damage, a phenomenon accompanied also by release of inflammatory cytokines and chemokines [126].

Astrocytic activation has been shown to occur in the visual cortex of the adult rats orally gavaged with Methyl-Hg (0.04 mg/kg/d) [127], and in the hippocampus of 30d-old rats prenatally exposed to Methyl-Hg (1−2 mg/kg in alternated days) [128]. Mercury exposure has been associated with the risk of ASD pathogenesis, and mercury accumulation within astrocytes, microglial activation, induction of oxidative stress, induction of proinflammatory pathways and mitochondrial dysfunctions are considered as some of the possible underlying neurotoxic mechanisms [129].

To our knowledge, our study is the first showing a direct impact of PCB138 on the percentage of hiPSC-derived neuronal and astrocytic cells in vitro. Other studies have reported about inhibitory effects of other PCBs or PCB mixture (e.g., Aroclor 1254) specifically on human oligodendrocytes [130], or in glial cancer cell models [131,132].

TCDD has been shown to promote proliferation of C6 astrocytes treated for 24 h with 10−50 nM [133], and to induce activation and migration of primary rat cortical astrocytes treated with 10^−4^-10^-3^ μM [134].

The increase of astrocytes experimentally observed in our culture system, upon exposure to BPA, CPF, Lead, PCB138, and in particular after mixture treatments, could be associated with induction of astrocyte-mediated neuroprotection triggered upon chemical exposure. Astrocytes are known to modulate BDNF production as a neuroprotective mechanism in response to chemicals [27] or stroke onset [135]. As commented in the previous section, some of the chemicals tested in this study can trigger BDNF increase. For instance, gestational exposure to BPA was found to upregulate BDNF expression in female rat offspring [79], exposure to CPF has been shown to upregulate BDNF levels in different experimental models [22,25,85,86], and increase of BDNF levels was reported in rats [80] and mice [81,82] exposed to Methyl-Hg.

### 4.3 Chemicals causing a decrease of synaptogenesis and neuronal electrical activity

BDE47 (at LOAEC-syn) resulted as the main trigger of decreased synapse number. Conversely, in our previous study we found that 14d treatment with less complex mixtures (containing either 3 or 6 chemicals) induced a general increase of synapses [31] (Fig. 10). It should be considered that our previous study did not include BDE47 in both ‘Similar MoA’ mixture and the ‘All’ mixture.

BDE47 resulted also as the most potent inducer of decreased electrical activity already after 7d treament, and treatment with both 5-Sim and 10-All mixtures perturbed this endpoint at levels comparable to those observed upon BDE47 single treatment.

In line with our results, prolonged exposure to BDE47 and its hydroxylated metabolites (e.g., 6OH-BDE47) has been shown to downregulate the maturation and function of embryonic rat cortical neurons, perturbing the expression of genes involved in neuronal activity [136]. These effects have been associated with inhibition of kinases (i.e., MEK-ERK signaling), leading to impairment of neuronal electrical activity, pre-synaptic functions, and axonal guidance [137].

### 4.4 Potential associations between observed perturbations of measured DNT endpoints and neurodevelopmental deficits in children

In recent years there has been a significant increase in the prevalence of neurodevelopmental disorders, as shown e.g., by the increase in ADHD, ASD, and lower IQ, and perinatal exposure to environmental chemicals is considered as one of the greatest risk factors in the development of these diseases [138,139]. However, mechanistic understanding of processes involved in these neurodevelopmental disorders was lacking. Making use of existing knowledge to explain a potential link between exposure to environmental chemicals and human diseases, the adverse outcome AOP concept has been developed, which identifies key events leading to a disease or an adverse outcome (https://aopwiki.org/). Dysregulation of BDNF levels or signalling, decrease of synaptogenesis and alterations in neuronal network formation and function represent common KEs described in different DNT-relevant AOPs, all leading to learning and memory impairment in children [[10], [11], [12], [13], [14], [15], [16], [17], [18], [19], [20], [21], [22], [23], [24], [25], [26], [27]].

Therefore, the changes in these key events, measured in our studies using in vitro assays based on human cells could be interpreted as potential DNT biomarkers, which might contribute to the development of neurodevelopmental disorders induced by exposure to mixture of chemicals present in the environment.

BDNF is involved in the promotion of neuronal protection, modulation of neurite outgrowth, excitability and synapse plasticity [74,75]. High BDNF levels have been found both in peripheral blood [140] and the frontal cortex [141] in ASD children, as confirmed by two meta-analyses [95,96]. However, both increased or decreased BDNF levels could lead to abnormal neuronal proliferation, differentiation or synapse formation resulting in altered neuronal network function, possibly leading to impairment of learning and memory processes, especially when brain structures, such as hippocampus or cortex [142], which are fundamental for memory formation, are involved.

In both our studies, we have observed an increase in the number of neuronal and glial cells. Interestingly, an increased neuronal cell number has been observed in the prefrontal cortex of ASD children (about 67 %) compared with healthy controls [143].

As discussed in the previous section, we observed a prominent increase in the percentage of GFAP^+^ astrocytes upon mixture treatment; such an increase may impact the formation of neuronal synapses, as astrocytes have recently been proposed to drive the refinement of neuronal circuits [144]. Furthermore, an aberrant increase of glial cells may have an impact on the onset of neuropsychiatric disorders [145]; increase of reactive glial cells may also contribute to loss of synaptic function, which may trigger ASD, as speculated by Li and coauthors [146].

### 4.5 The use of computational modelling to predict DNT endpoint sensitivity to mixture effects

By means of mathematical modelling, a synergistic effect on synaptogenesis-related proteins (PSD95 in particular) was established upon treatment with all tested mixtures (Fig. 8). Notably, BDE47 (present in both 5-Sim and 10-All mixtures) was considered as the main driver of mixture effects on SYP and on the number of synapses (upon treatment with both 5-Sim and 10-All at LOAEC-syn) (see stars in Fig. 8). On the other hand, for the other analysed endpoints (BDNF, neurite outgrowth, % neurons and astrocytes, all indicated by dashed black arrows in Fig. 8 and Supplementary Fig. 4, Fig. 5) the identification of synergism was not possible when the evaluation of the contribution of single chemicals revealed a response above the 5% threshold (TU > 1), even when the response elicited by the mixture was at least two-fold higher in magnitude than the most potent individual chemical. In these cases, an interactive effect was still hypothesized. Altogether, these data indicate that synaptogenesis may be the most sensitive DNT endpoint to chemical mixture effects, confirming our previously published observations [31]. Antagonistic effects, i.e., effects weaker than predicted, can be possibly investigated using the BMR method. However, this could be feasible under ideal experimental conditions, for instance when individual chemicals all cause the same type of effects (e.g., downregulation of a given endpoint or molecule), and the effects in the mixture follow the same trend and effect directionality, resulting weaker than predicted. However, in this DNT study, covering rather complex biological endpoints, depending on the tested chemicals and applied nominal concentration, both up- and down-regulation of analysed DNT endpoints could be observed, which makes distinguishing antagonistic effects challenging, if not impossible.

Furthermore, correlation plot analysis globally indicates that DNT effects do not always become greater simply by increasing the number of chemicals within a mixture, and that such effects may be diverse in both magnitude and direction depending on the analysed DNT endpoint (Fig. 10B). In particular, the overall DNT effects induced by the 3-Sim mixture are relatively different (in magnitude) from the effects induced by the 5-Sim mixture; on the contary, the 3-Diss mixture effects were comparable to the effects induced by the 5-Diss mixture. Also, the 5-Sim mixture effects are very similar to the effects induced by the 10-All mixture.

## 5 Conclusions

Altogether these results suggest that chemicals acting through similar MoA are the major drivers of DNT effects triggered upon exposure to complex chemical mixtures, as shown by the observed increase in BDNF levels, the decrease in synapses and neuronal network formation and function, and the increased proportion of neuronal cells and astrocytes observed in the applied human in vitro model. The use of hiPSC-derived NSCs undergoing differentiation toward neurons and astrocytes enables possible correlations to be established between perturbations of critical neurodevelopmental endpoints described as common KEs in the DNT-relevant AOP network and phenotypic features observed in childhood neurodevelopmental deficits. This demonstrates the utility of an in vitro test system, combined with mathematical modelling, for the assessment of DNT effects induced by chemical mixtures.

## Declaration of Competing Interest

The authors have no conflict of interests to declare.
