# Supplementary material for: Combining in vitro assays and mathematical modelling to study developmental neurotoxicity induced by chemical mixtures
Source: Reprod Toxicol. 2021 Oct;105:101–19. doi: 10.1016/j.reprotox.2021.08.007 (PMC8522961; doi:10.1016/j.reprotox.2021.08.007)
Supplement: Supplementary file 1 [file mmc1.docx]

**Supplementary Figures**

**Supplementary Figure 1. Best fitting curves for single chemicals**. Each panel represents the modeled response of single chemicals for all the selected DNT endpoints. To calculate the single chemical dose response effects for all the selected DNT endpoints, the following concentrations were tested: BDE47 (A) (i.e., 0.057, 0.198, 0.693, 2.426, 8.489, 13.58 μM), EtOH (B) (i.e., 82.16, 106.81, 138.85, 154.55, 170, 180.51, 187, 205.7, 226.27, 234.66, 256.9 mM), Vincl (C) (i.e., 0.08, 0.42, 2.10, 10.50, 52.50, 269.4 μM), and TCDD (D) (i.e, 17.30, 43.24, 108.10, 270.25, 675.63, 830 nM). The effect was estimated according to seven different mathematical models (i.e., Hill, Power, Linear, Polynomial 2, Exponential 2, Exponential 3, Exponential 4, and Exponential 5) by using the BMDExpress.2 open access software (<https://github.com/auerbachs/BMDExpress-2/wiki>). The best-fitting curve across the range of concentration tested is represented in the figure as percentage of response compared to the solvent control (0.1% DMSO).

**
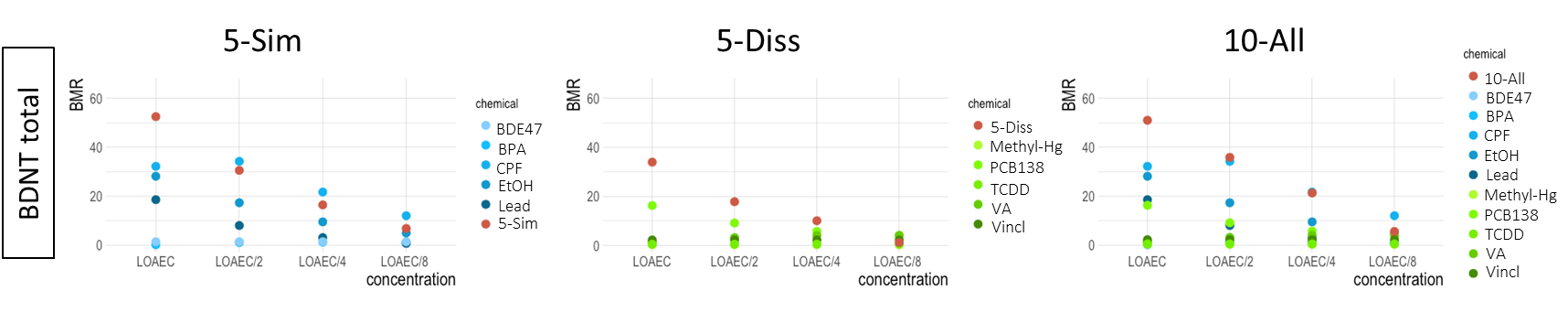
**

**Supplementary Figure 2.** Bench mark responses (BMR) for total BDNF levels. The absolute BMR value of single chemicals, calculated considering their concentrations used in the mixtures ('5-Sim', '5-Diss', and '10-All'), are plotted and compared with the absolute percentage of response observed experimentally in the mixtures for this specific DNT endpoint (normalised to solvent control). For this endpoint, the evaluation of the contribution of single chemicals revealed a response above the 5% threshold (TU > 1). As the response elicited by the mixture was not at least two-folds of magnitude higher than the most potent individual chemical contribution, no interactive mixture effect can be hypothesized for this DNT endpoint.

**Supplementary Figure 3.** **Examples of combined effects after 14d treatment with mixtures.** NSCs were differentiated for 7 DIV, and then treated for 14d with single chemicals and three types of mixtures as described in Figure 3 legend. (A) Graph reports neurite length (black), number of neurites/neuron (dashed grey) and number of branch points/neurite (violet) analyzed upon treatment with LOAEC/4-neu specific concentrations (see Table 2). (B) Graph reports percentage of β-III-tubulin^+^ (red) and GFAP^+^ cells (green), analyzed upon treatment with LOAEC/4-neu specific concentrations (see Table 2). (C) Graph shows total levels of SYP (green), PSD95 (red), and number of overlapping SYP/PSD95 puncta (synapses, orange) analyzed upon treatment with LOAEC/2-syn specific concentrations (see Table 2). All samples were normalised to solvent control (0.1% DMSO, Ctr) at the respective time point. Data are represented as mean ± S.E.M. of 3-4 biological replicates.

**
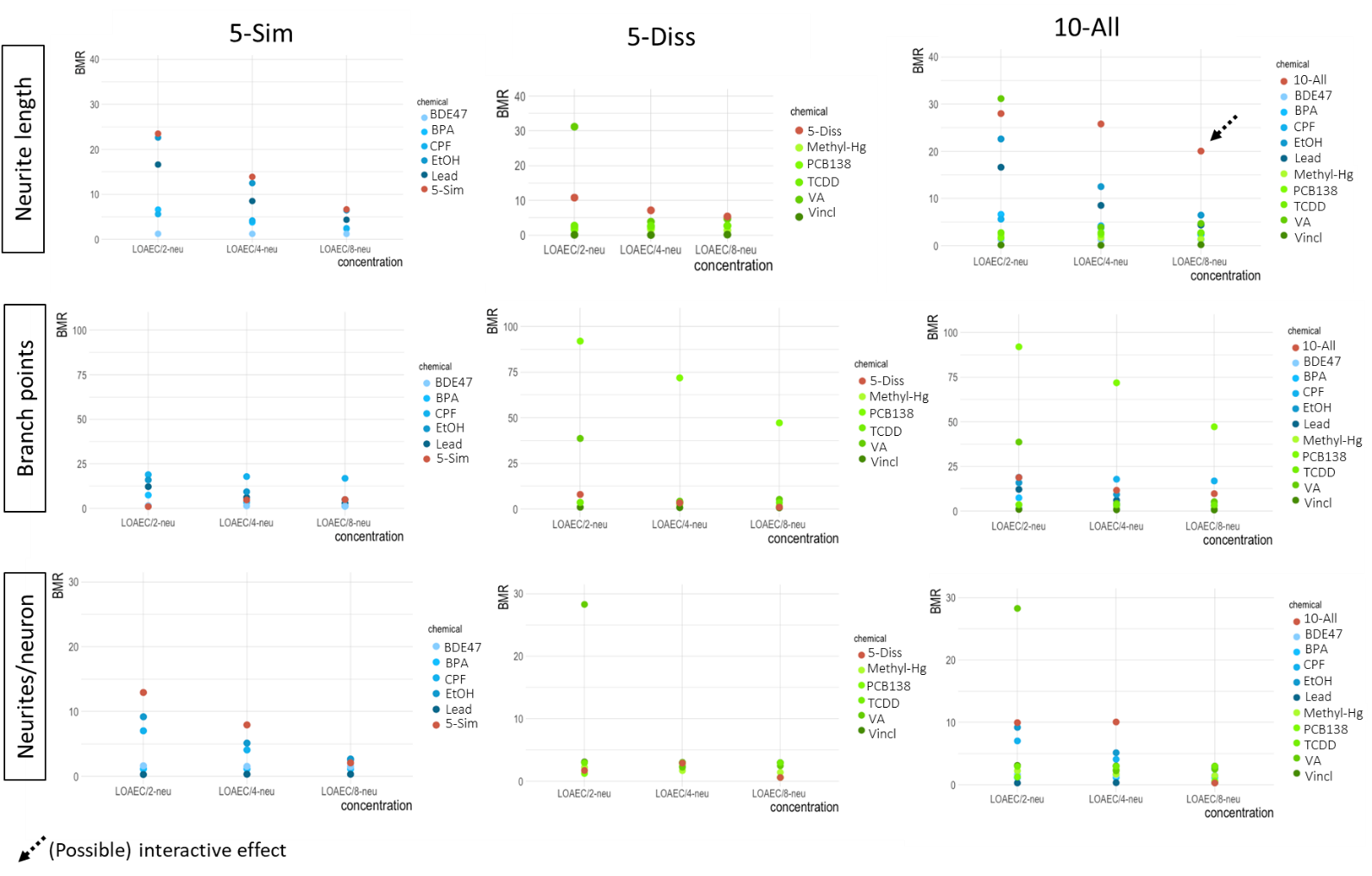
**

**Supplementary Figure 4.** Bench mark responses (BMR) for neurite outgrowth. The absolute BMR value of single chemicals, calculated considering their concentrations used in the mixtures ('5-Sim', '5-Diss', and '10-All'), are plotted and compared with the absolute percentage of response observed experimentally in the mixtures for each DNT endpoint (normalised to solvent control). Although, for these endpoints the evaluation of the contribution of single chemicals revealed a response above the 5% threshold (TU > 1), an interactive mixture effect can be hypothesized when the response elicited by the mixture was at least two-folds of magnitude higher than the most potent individual chemical contribution (indicated by the black dashed arrows).

**
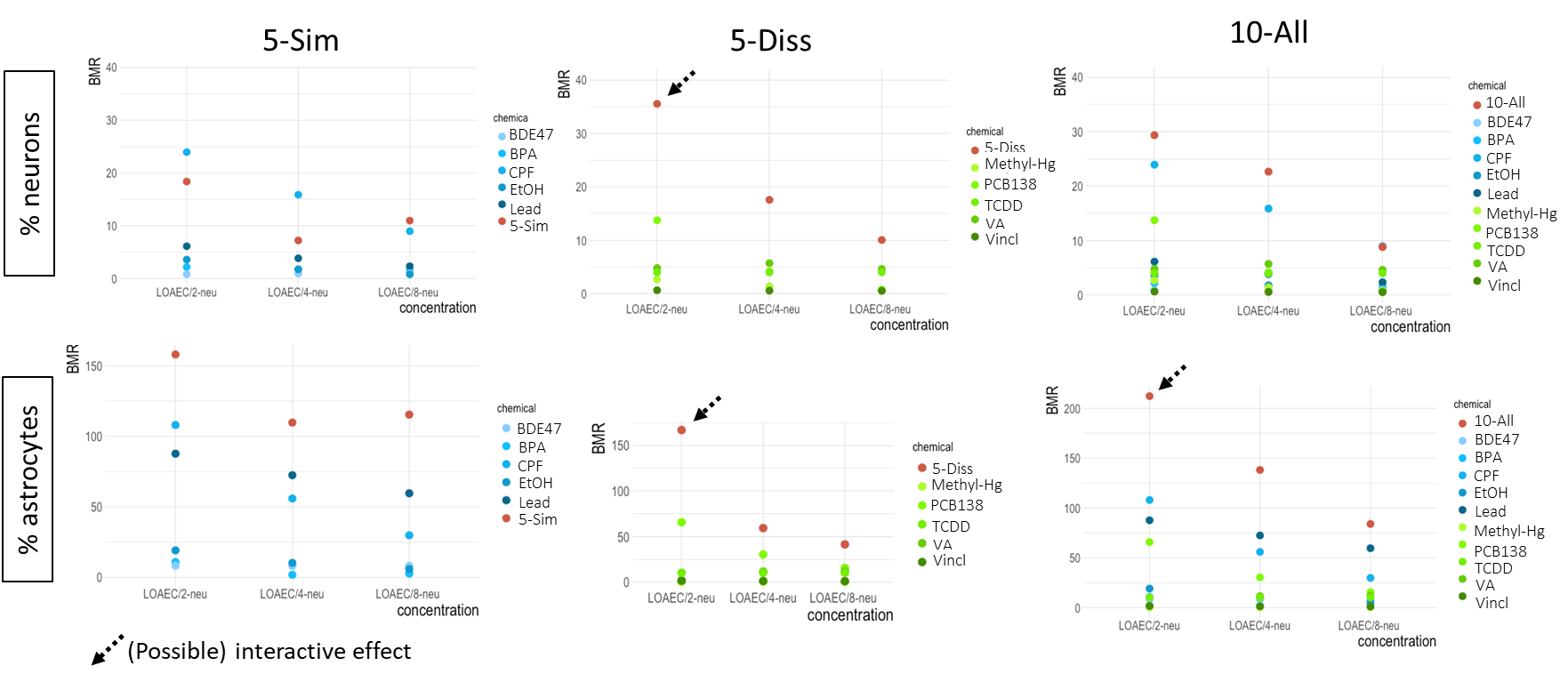
**

**Supplementary Figure 5.** Bench mark responses (BMR) for percentage of neurons and astrocytes. The absolute BMR value of single chemicals, calculated considering their concentrations used in the mixtures ('5-Sim', '5-Diss', and '10-All'), are plotted and compared with the absolute percentage of response observed experimentally in the mixtures for each DNT endpoint (normalised to solvent control). Although, for these endpoints the evaluation of the contribution of single chemicals revealed a response above the 5% threshold (TU > 1), an interactive mixture effect can be hypothesized when the response elicited by the mixture was at least two-folds of magnitude higher than the most potent individual chemical contribution (indicated by the black dashed arrows).


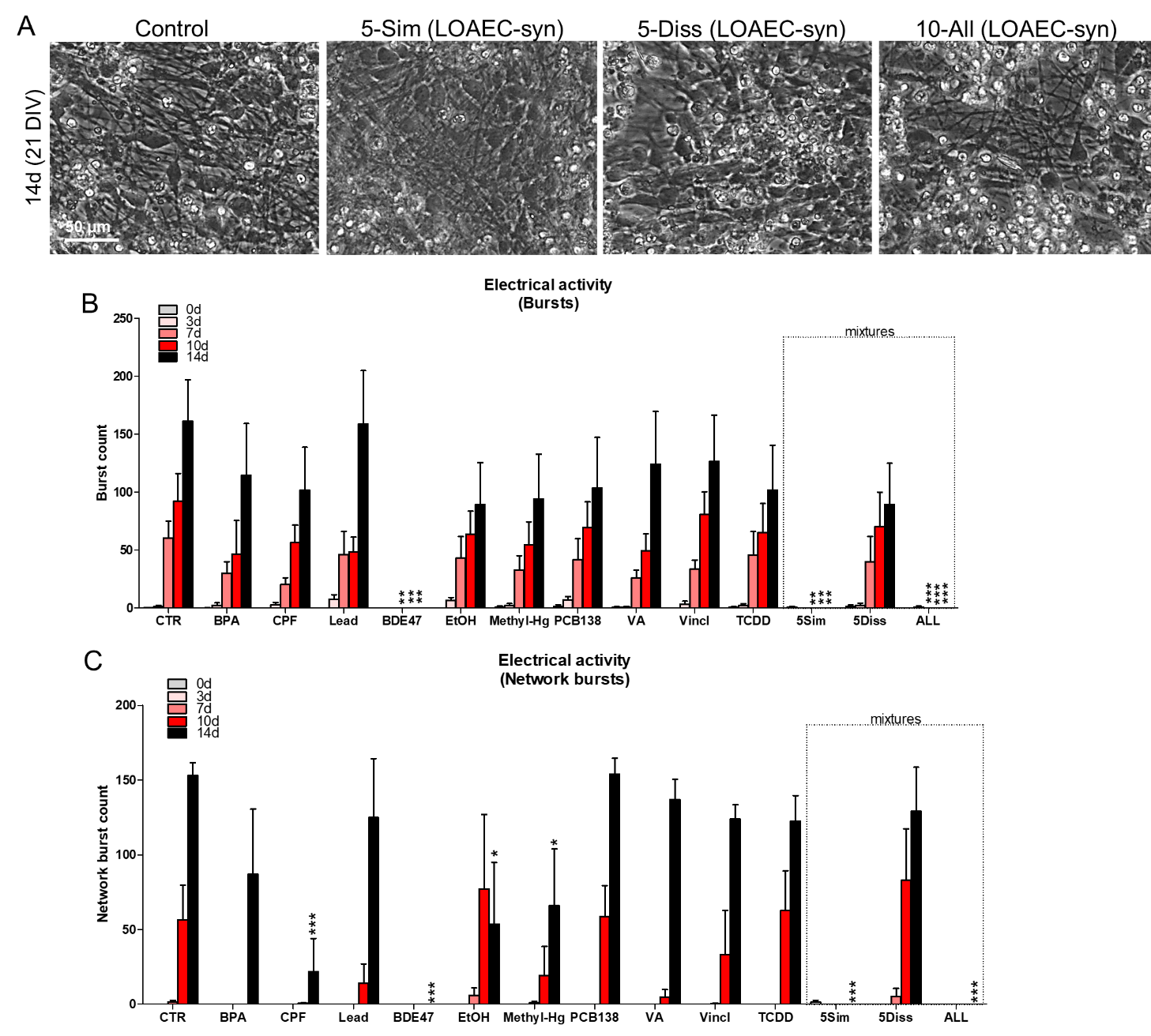


**Supplementary Figure 6.** **Mixture effects on electrical activity.** Representative phase contrast images of NSC-derived neuronal cells cultured on a multi-well MEA 24-well plate treated for 14d with 5-Sim, 5-Diss and 10-All mixtures at the concentration of LOAEC-syn or solvent control (0.1% DMSO). (B, C) Graphs report total burst count (B) and network burst count (C) measured during 5 min recording, and analyzed after 0, 3, 7, 10 and 14d treatment with either single chemicals or mixtures (at LOAEC-syn concentration) or solvent control (Ctr, 0.1% DMSO). Data are represented as mean ± S.E.M. of 3-4 biological replicates.
