# Supplementary material for: Combining in vitro assays and mathematical modelling to study developmental neurotoxicity induced by chemical mixtures
Source: Reprod Toxicol. 2021 Oct;105:101–19. doi: 10.1016/j.reprotox.2021.08.007 (PMC8522961; doi:10.1016/j.reprotox.2021.08.007)
Supplement: Supplementary file 2 [file mmc2.docx]

**Supplementary Materials and Methods: VCBA**

*Manuscript title: Combining in vitro assays and mathematical modelling to study developmental neurotoxicity induced by chemical mixtures*

**Virtual Cell Based Assay**

To obtain the free concentration that can perturb and affect the cell system in an experimental setup, it is of interest to measure the chemical distribution in each fraction (i.e., medium, plastic, headspace, and cell). However, in some cases, this is not feasible and to save time, prediction models to estimate the partitioning of the chemical in the well can be used. Recently, Proença et al. (Proença et al. 2021) provided an overview of such prediction models, among them the Virtual Cell Based Assay (VCBA). The VCBA is a mathematical model that was developed during the SEURAT-1 consortium -COSMOS project - at the EC Joint Research Centre (JRC) (Comenges et al. 2017). The model represents the distribution of a chemical in a well and predicts the partitioning to plastic, cellular and extracellular components, evaporation, and free concentration. The free concentration will then distribute within the cell (as well as predictions to the cell lipid, protein and water fractions). More description of the VCBA model can be found in (Comenges et al. 2017).

**Methodology**

The VCBA model was developed in Matlab by (Zaldívar Comenges et al. 2011; Zaldívar Comenges et al. 2010), it was extensively revised and rewritten in R by (Proença et al. 2019). The R version was used in the current exercise. R code was run in R studio Version 1.2.1335 (2009-2019 RStudio, Inc.version). The VCBA R code used can be shared upon request by email to [JRC-F3-ENQUIRIES@ec.europa.eu](mailto:JRC-F3-ENQUIRIES@ec.europa.eu).

Physico-chemical input parameters (Table SM1) to run the model were searched in the following databases during the period of 15^th^ -20^th^ of July 2021: Chemspider (<http://www.chemspider.com/> ) and Pubchem (<https://pubchem.ncbi.nlm.nih.gov/>). EpiSuite (version EPIWEB 4.1) was used to calculate Lead and Methyl-Hg parameters for air and water degradation.

**Table SM1. Physico-chemical input parameters to parametrise the VCBA model.**

| Chemicals | BPA | CPF | Lead | BDE-47 | EtOH | Methyl-Hg | PCB-138 | VA | Vincl | TCDD |
| --- | --- | --- | --- | --- | --- | --- | --- | --- | --- | --- |
| Mlogkow | 3,32 | 4,96 | 0,73 | 7,39 | -0,31 | 0,41 | 7,44 | 2,75 | 3,1 | 7,01 |
| Mkdeccomp #Water  Degradation rate in s-1 | 2,14E-07 | 4,45  E-08 | 2,14E-07 | 4,46E-08 | 9,26E-07 | 2,14E-07 | 4,46E-08 | 5,35E-07 | 1,34E-07 | 4,46E-08 |
| Mkdecacomp#Air  Degradation in s-1 | 6,42E-05 | 6,42E-05 | 1,93E-09 | 7,52E-07 | 2,45E-06 | 6,13E-06 | 1,23E-07 | 6,13E-06 | 2,59E-05 | 5,65E-07 |
| MMWcomp#Compound Molecular Weight g/mol | 228,29 | 350,59 | 278 | 486 | 46,1 | 252 | 360,88 | 144,22 | 286,11 | 321,97 |
| MSvcomp  #atomic diffusion | 220,14 | 348,56 | 174,96 | 174,96 | 51,77 | 245,13 | 282,52 | 174,64 | 256,75 | 254,48 |
| MH37 #Henry´s constant at 37 C Pa*m3/mol | 9,28E-07 | 0,297 | 3,72E-02 | 3,01E-01 | 0,507 | 0,367 | 6,94 | 0,304 | 1,35E-03 | 3,58E-01 |
| Mvb  #Molar volume | 200 | 236,8 | 2,22E+02 | 198,2 | 59,1 | 201,2 | 199,7 | 194,8 | 167,5 | 171,85 |

Based on the cell type and experimental set up, the following input parameters to run the VCBA were selected (Table SM2). Considering the lack of serum in the medium, “serum well partition” was set to zero; lipid and protein component of matrigel were also set to zero since the experimental results published by (Di Consiglio et al. 2020) found that sequestration of chemicals by matrigel can be considered as negligible. Therefore, it was concluded that extracellular binding in the medium is negligible and only plastic and cell components may influence the free concentration.

The following assumptions were made on the cell characterisation to run the VCBA model:

1. the cell model was considered similar to fibroblasts in cell fractions content (water, protein, lipid);
2. metabolic competence in formation of metabolite(s) was considered negligible;
3. cells undergoing differentiation were considered to not undergo cell division;
4. active cellular uptake was considered to not take place.

The well geometry module followed the size of 96 well plates (table SM2).

Finally, the applicability domain of the VCBA is to organic chemicals, so the prediction of the two organometallic compounds (i.e., Methyl-Hg and Lead) can be questionable. Moreover, the VCBA model does not take into account chemical ionisation.

**Table SM2. Model input data for experimental set up**

(Medium composition, well plate geometry, cell line type)

|  | Parameter | Unit |
| --- | --- | --- |
| Cell type | NSCs undergoing differentiation |  |
| Aqueous fraction | 0.614 |  |
| Protein fraction | 0.244 |  |
| Lipid fraction | 0.142 |  |
| Cell growth and division | NO |  |
| Metabolic competence | Negligible |  |
| Cell number | 7000 | cells/well |
| Well Geometry | 96 | plate |
| Volume of medium | 0.150 | mL |
| Top internal radius | 3.425·10^−3^ | m |
| Bottom internal diameter | 3.175·10^−3^ | m |
| Depth | 10.76·10^−3^ | m |
| Bottom area | 3.16·10^–5^ | m^2^ |
| Volume of well | 0.36 | mL |
| Headspace volume | 2.6·10^−7^ | (VH)(m^3^) |
| Medium composition |  |  |
| %protein (Matrigel)* | 0 | % |
| %Lipid (Matrigel)* | 0 | % |
| Serum | Not present put to 0 |  |
| Exposure | Single |  |
| *protein in matrigel max 5% for CPF = negligible (Di Consiglio et al., 2021) | | |

Table SM3 reports the experimental nominal concentrations used in this study in M (molar) unit, which were used to run the model. When converting concentrations in M unit, it was taken into account that the concentrations of Ethanol were in mM, while the other chemicals were in the µM range.

**Table SM3. Experimental nominal concentrations (in Molar) for each chemical measured in the cell system for the different DNT endpoints** (see also Table 2 in the main manuscript).

| (M) | BPA | CPF | Lead | BDE-47 | EtOH | Methyl-Hg | PCB-138 | VA | Vincl | TCDD |
| --- | --- | --- | --- | --- | --- | --- | --- | --- | --- | --- |
| BDNF | 1,27E-05 | 3,71E-05 | 1,46E-06 | 2,00E-07 | 1,07E-01 | 1,30E-07 | 3,53E-06 | 2,10E-04 | 4,20E-07 | 6,00E-07 |
|  | 6,37E-06 | 1,86E-05 | 7,30E-07 | 1,00E-07 | 5,34E-02 | 7,00E-08 | 1,77E-06 | 1,05E-04 | 2,10E-07 | 3,00E-07 |
|  | 3,19E-06 | 9,28E-06 | 3,70E-07 | 5,00E-08 | 2,67E-02 | 3,00E-08 | 8,8E-07 | 5,25E-05 | 1,10E-07 | 1,50E-07 |
|  | 1,59E-06 | 4,64E-06 | 1,80E-07 | 2,00E-08 | 1,34E-02 | 1,70E-08 | 4,4E-07 | 2,63E-05 | 5,00E-08 | 8,00E-08 |
| Neurite Outgrowth | 1,27E-05 | 3,71E-05 | 1,46E-06 | 2,00E-07 | 1,70E-01 | 1,30E-07 | 1,19E-05 | 4,20E-04 | 5,31E-06 | 4,00E-08 |
|  | 6,37E-06 | 1,86E-05 | 7,30E-07 | 1,00E-07 | 8,50E-02 | 7,00E-08 | 5,93E-06 | 2,10E-04 | 2,66E-06 | 2,00E-08 |
|  | 3,19E-06 | 9,28E-06 | 3,70E-07 | 5,00E-08 | 4,25E-02 | 3,00E-08 | 2,97E-06 | 1,05E-04 | 1,33E-06 | 1,00E-08 |
|  | 1,59E-06 | 4,64E-06 | 1,80E-07 | 2,00E-08 | 2,13E-02 | 1,60E-08 | 1,48E-06 | 5,25E-05 | 6,60E-07 | 5,00E-09 |
| Synaptogenesis | 1,27E-05 | 2,1E-05 | 7,30E-09 | 1,76E-05 | 1,07E-01 | 5,00E-08 | 6,00E-08 | 2,10E-06 | 4,20E-07 | 6,00E-07 |
|  | 6,37E-06 | 1,05E-05 | 3,70E-09 | 8,81E-06 | 5,34E-02 | 2,50E-08 | 3,00E-08 | 1,05E-06 | 2,10E-07 | 3,00E-07 |
|  | 3,19E-06 | 5,25E-06 | 1,80E-09 | 4,41E-06 | 2,67E-02 | 1,3E-08 | 1,50E-08 | 5,3E-07 | 1,10E-07 | 1,5,E-07 |
|  | 1,59E-06 | 2,63E-06 | 9,00E-10 | 2,20E-06 | 1,34E-02 | 6,00E-09 | 7,40E-09 | 2,60E-07 | 5,00E-08 | 8,00E-08 |

The summarised results (in percentage) are reported in table SM4, while the raw prediction data in molar (M) are shown in table SM5. The results showed that between 85 to 96% of chemicals were available to the cells (either as free concentration in medium, or sequestered by cellular lipids).

The concentrations of chemicals that migrate to the plastic ranged between 0 – 5% for the most lipophilic chemicals. Fraction evaporated to headspace for all the 10 chemicals was negligible. As highlighted in the methodology section the protein and lipid in medium /serum were set to zero.

**Table SM4. Summary of the percentages of distribution of the 10 chemicals in different fractions calculated by the VCBA model.**

| Chemical name | LogP | Nominal concentration calculated (exp dilution serie) | Concentration in medium - Mass balance - % | Dissolved concentration not bound - % | Concentration to protein - % | Concentration to lipid - (Cell membrane) % | Concentration to plastic - % | Concentration in headspace (evaporated) - % | Fraction available to the cells -% |
| --- | --- | --- | --- | --- | --- | --- | --- | --- | --- |
| CPF | 4,96 | 100 | 97,2 | 0,8 | 0 | 93 | 3,6 | 0 | 93,6 |
| BPA | 3,32 | 100 | 97,3 | 44,2 | 0 | 48 | 5,3 | 0 | 91,9 |
| EtOH | -0,31 | 100 | 85,2 | 85,2 | 0 | 0 | 0 | 0 | 85,2 |
| Methyl-Hg | 0,41 | 100 | 96,3 | 96,3 | 0 | 0 | 0 | 0 | 96,3 |
| VA | 2,75 | 100 | 92,0 | 74,1 | 0 | 15 | 2 | 0 | 89,5 |
| Lead | 0,73 | 100 | 96,4 | 96,3 | 0 | 0 | 0 | 0 | 96,3 |
| BDE-47 | 7,39 | 100 | 94,8 | 0,0 | 0 | 94 | 1 | 0 | 94,0 |
| PCB-138 | 7,44 | 100 | 94,7 | 0,0 | 0 | 93 | 1 | 0 | 93,5 |
| Vincl | 3,1 | 100 | 98,0 | 59,5 | 0 | 34 | 4 | 0 | 93,6 |
| TCDD | 7,01 | 100 | 95,2 | 0,0 | 0 | 94 | 1 | 0 | 94,2 |

As an evaluation step, model predictions were analysed against the *in vitro* experiments carried out by (Di Consiglio et al. 2020). In this work, Di Consiglio and co-workers measured the distribution of CPF in cells, medium, plastic and matrigel. In particular, the fraction predicted by the VCBA model that migrated to the plastic was 3.6%; this fraction is in line with the range of 0-8.4% measured *in vitro* by (Di Consiglio et al. 2020). For matrigel, the sequestration of CPF was found to be negligible.

An additional evaluation was done using predicted results published in (Proença et al. 2019). In this work, using similar settings, Ethanol partitioned in the dissolved (free) phase (83%) and in proteins (2-5%). The results from the partitioning using the VCBA was 85% fraction as free concentration and 0% to protein (since in this work, the extracellular protein fraction was set to zero).

While validation based only on these two chemicals cannot hold for all the other chemicals tested in this study, it shows that the VCBA model is in line with what was reported in previous studies, and can represent a promising tool to extrapolate and correct for artefacts in *in vitro* systems, enabling to predict the chemical fraction that can be available to the cells. This is of particular interest when performing reverse dosimetry or quantitative *in vitro-to-in vivo* extrapolation. Additional experiments should be performed to validate the VCBA model predictions.

**Table SM5. VCBA predicted chemical concentrations in the well (in Molar), considering the nominal concentrations used in the study** (see also Table 2 in the manuscript)

| Chemical name | Nominal concentration calculated (exp dilution serie) | Concentration in medium - Mass balance - in M | Dissolved concentration not bound - in M | Concentration bound to protein in M (Serum ) | Concentration bound to lipid (Cell membrane) in M | Concentration bound to plastic in M | Concentration in headspace (evaporated) in M | LogP (as check point) |
| --- | --- | --- | --- | --- | --- | --- | --- | --- |
| CPF | 3,71E-05 | 3,61E-05 | 2,85E-07 | 0 | 3,45E-05 | 1,34E-06 | 3,34E-11 | 4,96 |
| CPF | 1,86E-05 | 1,80E-05 | 1,42E-07 | 0 | 1,72E-05 | 6,69E-07 | 1,67E-11 | 4,96 |
| CPF | 9,28E-06 | 9,02E-06 | 7,12E-08 | 0 | 8,62E-06 | 3,35E-07 | 8,37E-12 | 4,96 |
| CPF | 4,64E-06 | 4,51E-06 | 3,56E-08 | 0 | 4,31E-06 | 1,67E-07 | 4,18E-12 | 4,96 |
| CPF | 2,10E-05 | 2,04E-05 | 1,61E-07 | 0 | 1,95E-05 | 7,58E-07 | 1,89E-11 | 4,96 |
| CPF | 1,05E-05 | 1,02E-05 | 8,07E-08 | 0 | 9,76E-06 | 3,79E-07 | 9,47E-12 | 4,96 |
| CPF | 5,25E-06 | 5,11E-06 | 4,03E-08 | 0 | 4,88E-06 | 1,89E-07 | 4,73E-12 | 4,96 |
| CPF | 2,63E-06 | 2,56E-06 | 2,02E-08 | 0 | 2,44E-06 | 9,49E-08 | 2,37E-12 | 4,96 |
|  |  |  |  |  |  |  |  |  |
| Chemical name | **Nominal concentration calculated (exp dilution serie)** | **Concentration in medium - Mass balance - in M** | **Dissolved concentration not bound - in M** | **Concentration bound to protein in M (Serum )** | **Concentration bound to lipid (Cell membrane) in M** | **Concentration bound to plastic in M** | **Concentration in headspace (evaporated) in M** | **LogP (as check point)** |
| BPA | 1,27E-05 | 1,24E-05 | 5,64E-06 | 0 | 6,08E-06 | 6,79E-07 | 2,05E-15 | 3,32 |
| BPA | 6,37E-06 | 6,20E-06 | 2,82E-06 | 0 | 3,04E-06 | 3,40E-07 | 1,02E-15 | 3,32 |
| BPA | 3,19E-06 | 3,10E-06 | 1,41E-06 | 0 | 1,52E-06 | 1,70E-07 | 5,13E-16 | 3,32 |
| BPA | 1,59E-06 | 1,55E-06 | 7,03E-07 | 0 | 7,58E-07 | 8,48E-08 | 2,56E-16 | 3,32 |
|  |  |  |  |  |  |  |  |  |
| Chemical name | **Nominal concentration calculated (exp dilution serie)** | **Concentration in medium - Mass balance - in M** | **Dissolved concentration not bound - in M** | **Concentration bound to protein in M (Serum )** | **Concentration bound to lipid (Cell membrane) in M** | **Concentration bound to plastic in M** | **Concentration in headspace (evaporated) in M** | **LogP (as check point)** |
| EtOH | 0,10681 | 0,090980302 | 0,090974154 | 0 | 2,85E-06 | 3,30E-06 | 1,79E-05 | -0,31 |
| EtOH | 0,053405 | 0,045490151 | 0,045487077 | 0 | 1,42E-06 | 1,65E-06 | 8,94E-06 | -0,31 |
| EtOH | 0,026703 | 0,022745501 | 0,022743964 | 0 | 7,11E-07 | 8,26E-07 | 4,47E-06 | -0,31 |
| EtOH | 0,013351 | 0,011372325 | 0,011371556 | 0 | 3,56E-07 | 4,13E-07 | 2,24E-06 | -0,31 |
| EtOH | 0,17 | 0,144805275 | 0,144795489 | 0 | 4,53E-06 | 5,26E-06 | 2,85E-05 | -0,31 |
| EtOH | 0,085 | 0,072402637 | 0,072397744 | 0 | 2,26E-06 | 2,63E-06 | 1,42E-05 | -0,31 |
| EtOH | 0,0425 | 0,036201319 | 0,036198872 | 0 | 1,13E-06 | 1,31E-06 | 7,12E-06 | -0,31 |
| EtOH | 0,02125 | 0,018100659 | 0,018099436 | 0 | 5,66E-07 | 6,57E-07 | 3,56E-06 | -0,31 |
|  |  |  |  |  |  |  |  |  |
| Chemical name | **Nominal concentration calculated (exp dilution serie)** | **Concentration in medium - Mass balance - in M** | **Dissolved concentration not bound - in M** | **Concentration bound to protein in M (Serum )** | **Concentration bound to lipid (Cell membrane) in M** | **Concentration bound to plastic in M** | **Concentration in headspace (evaporated) in M** | **LogP (as check point)** |
| Methyl-Hg | 1,30E-07 | 1,25E-07 | 1,25E-07 | 0 | 3,11E-11 | 2,27E-11 | 1,78E-11 | 0,41 |
| Methyl-Hg | 7,00E-08 | 6,74E-08 | 6,74E-08 | 0 | 1,67E-11 | 1,22E-11 | 9,59E-12 | 0,41 |
| Methyl-Hg | 3,00E-08 | 2,89E-08 | 2,89E-08 | 0 | 7,18E-12 | 5,24E-12 | 4,11E-12 | 0,41 |
| Methyl-Hg | 1,70E-08 | 1,64E-08 | 1,64E-08 | 0 | 4,07E-12 | 2,97E-12 | 2,33E-12 | 0,41 |
| Methyl-Hg | 5,00E-08 | 4,82E-08 | 4,81E-08 | 0 | 1,20E-11 | 8,73E-12 | 6,85E-12 | 0,41 |
| Methyl-Hg | 2,50E-08 | 2,41E-08 | 2,41E-08 | 0 | 5,98E-12 | 4,36E-12 | 3,43E-12 | 0,41 |
| Methyl-Hg | 1,30E-08 | 1,25E-08 | 1,25E-08 | 0 | 3,11E-12 | 2,27E-12 | 1,78E-12 | 0,41 |
| Methyl-Hg | 6,00E-09 | 5,78E-09 | 5,78E-09 | 0 | 1,44E-12 | 1,05E-12 | 8,22E-13 | 0,41 |
|  |  |  |  |  |  |  |  |  |
| Chemical name | **Nominal concentration calculated (exp dilution serie)** | **Concentration in medium - Mass balance - in M** | **Dissolved concentration not bound - in M** | **Concentration bound to protein in M (Serum )** | **Concentration bound to lipid (Cell membrane) in M** | **Concentration bound to plastic in M** | **Concentration in headspace (evaporated) in M** | **LogP (as check point)** |
| VA | 0,00021 | 0,000193259 | 0,000155505 | 0 | 3,25E-05 | 5,25E-06 | 1,84E-08 | 2,75 |
| VA | 0,000105 | 9,66E-05 | 7,78E-05 | 0 | 1,63E-05 | 2,62E-06 | 9,20E-09 | 2,75 |
| VA | 5,25E-05 | 4,83E-05 | 3,89E-05 | 0 | 8,13E-06 | 1,31E-06 | 4,60E-09 | 2,75 |
| VA | 2,63E-05 | 2,42E-05 | 1,94E-05 | 0 | 4,06E-06 | 6,56E-07 | 2,30E-09 | 2,75 |
| VA | 0,00042 | 0,000386517 | 0,000311011 | 0 | 6,50E-05 | 1,05E-05 | 3,68E-08 | 2,75 |
| VA | 0,00021 | 0,000193259 | 0,000155505 | 0 | 3,25E-05 | 5,25E-06 | 1,84E-08 | 2,75 |
| VA | 0,000105 | 9,66E-05 | 7,78E-05 | 0 | 1,63E-05 | 2,62E-06 | 9,20E-09 | 2,75 |
| VA | 5,25E-05 | 4,83E-05 | 3,89E-05 | 0 | 8,13E-06 | 1,31E-06 | 4,60E-09 | 2,75 |
| VA | 2,10E-06 | 1,93E-06 | 1,56E-06 | 0 | 3,25E-07 | 5,25E-08 | 1,84E-10 | 2,75 |
| VA | 1,05E-06 | 9,66E-07 | 7,78E-07 | 0 | 1,63E-07 | 2,62E-08 | 9,20E-11 | 2,75 |
| VA | 5,30E-07 | 4,88E-07 | 3,92E-07 | 0 | 8,20E-08 | 1,32E-08 | 4,64E-11 | 2,75 |
| VA | 2,60E-07 | 2,39E-07 | 1,93E-07 | 0 | 4,02E-08 | 6,50E-09 | 2,28E-11 | 2,75 |
|  |  |  |  |  |  |  |  |  |
| Chemical ID/Name | **Nominal concentration calculated (exp dilution serie)** | **Concentration in medium - Mass balance - in M** | **Dissolved concentration not bound - in M** | **Concentration bound to protein in M (Serum )** | **Concentration bound to lipid (Cell membrane) in M** | **Concentration bound to plastic in M** | **Concentration in headspace (evaporated) in M** | **LogP (as check point)** |
| Lead | 1,46E-06 | 1,41E-06 | 1,41E-06 | 0 | 8,77E-10 | 5,21E-10 | 2,03E-11 | 0,73 |
| Lead | 7,30E-07 | 7,03E-07 | 7,03E-07 | 0 | 4,39E-10 | 2,60E-10 | 1,01E-11 | 0,73 |
| Lead | 3,70E-07 | 3,57E-07 | 3,56E-07 | 0 | 2,22E-10 | 1,32E-10 | 5,14E-12 | 0,73 |
| Lead | 1,80E-07 | 1,73E-07 | 1,73E-07 | 0 | 1,08E-10 | 6,42E-11 | 2,50E-12 | 0,73 |
| Lead | 7,30E-09 | 7,03E-09 | 7,03E-09 | 0 | 4,39E-12 | 2,60E-12 | 1,01E-13 | 0,73 |
| Lead | 3,70E-09 | 3,57E-09 | 3,56E-09 | 0 | 2,22E-12 | 1,32E-12 | 5,14E-14 | 0,73 |
| Lead | 1,80E-09 | 1,73E-09 | 1,73E-09 | 0 | 1,08E-12 | 6,42E-13 | 2,50E-14 | 0,73 |
| Lead | 9,00E-10 | 8,67E-10 | 8,66E-10 | 0 | 5,41E-13 | 3,21E-13 | 1,25E-14 | 0,73 |
|  |  |  |  |  |  |  |  |  |
| Chemical ID/Name | **Nominal concentration calculated (exp dilution serie)** | **Concentration in medium - Mass balance - in M** | **Dissolved concentration not bound - in M** | **Concentration bound to protein in M (Serum )** | **Concentration bound to lipid (Cell membrane) in M** | **Concentration bound to plastic in M** | **Concentration in headspace (evaporated) in M** | **LogP (as check point)** |
| BDE-47 | 2,00E-07 | 1,90E-07 | 1,43E-12 | 0 | 1,88E-07 | 1,52E-09 | 1,70E-16 | 7,39 |
| BDE-47 | 1,00E-07 | 9,48E-08 | 7,13E-13 | 0 | 9,40E-08 | 7,62E-10 | 8,50E-17 | 7,39 |
| BDE-47 | 5,00E-08 | 4,74E-08 | 3,57E-13 | 0 | 4,70E-08 | 3,81E-10 | 4,25E-17 | 7,39 |
| BDE-47 | 2,00E-08 | 1,90E-08 | 1,43E-13 | 0 | 1,88E-08 | 1,52E-10 | 1,70E-17 | 7,39 |
| BDE-47 | 1,76E-05 | 1,67E-05 | 1,26E-10 | 0 | 1,66E-05 | 1,34E-07 | 1,50E-14 | 7,39 |
| BDE-47 | 8,81E-06 | 8,35E-06 | 6,28E-11 | 0 | 8,28E-06 | 6,72E-08 | 7,48E-15 | 7,39 |
| BDE-47 | 4,41E-06 | 4,18E-06 | 3,14E-11 | 0 | 4,15E-06 | 3,36E-08 | 3,75E-15 | 7,39 |
| BDE-47 | 2,20E-06 | 2,09E-06 | 1,57E-11 | 0 | 2,07E-06 | 1,68E-08 | 1,87E-15 | 7,39 |
|  |  |  |  |  |  |  |  |  |
| Chemical name | **Nominal concentration calculated (exp dilution serie)** | **Concentration in medium - Mass balance - in M** | **Dissolved concentration not bound - in M** | **Concentration bound to protein in M (Serum )** | **Concentration bound to lipid (Cell membrane) in M** | **Concentration bound to plastic in M** | **Concentration in headspace (evaporated) in M** | **LogP (as check point)** |
| PCB-138 | 3,53E-06 | 3,34E-06 | 2,17E-11 | 1,96E-08 | 3,30E-06 | 2,59E-08 | 5,95E-14 | 7,44 |
| PCB-138 | 1,77E-06 | 1,68E-06 | 1,09E-11 | 9,85E-09 | 1,65E-06 | 1,30E-08 | 2,98E-14 | 7,44 |
| PCB-138 | 8,80E-07 | 8,34E-07 | 5,40E-12 | 4,90E-09 | 8,22E-07 | 6,45E-09 | 1,48E-14 | 7,44 |
| PCB-138 | 4,40E-07 | 4,17E-07 | 2,70E-12 | 2,45E-09 | 4,11E-07 | 3,23E-09 | 7,42E-15 | 7,44 |
| PCB-138 | 1,19E-05 | 1,13E-05 | 7,30E-11 | 6,62E-08 | 1,11E-05 | 8,73E-08 | 2,01E-13 | 7,44 |
| PCB-138 | 5,93E-06 | 5,62E-06 | 3,64E-11 | 3,30E-08 | 5,54E-06 | 4,35E-08 | 1,00E-13 | 7,44 |
| PCB-138 | 2,97E-06 | 2,81E-06 | 1,82E-11 | 1,65E-08 | 2,78E-06 | 2,18E-08 | 5,01E-14 | 7,44 |
| PCB-138 | 1,48E-06 | 1,40E-06 | 9,08E-12 | 8,24E-09 | 1,38E-06 | 1,09E-08 | 2,49E-14 | 7,44 |
|  |  |  |  |  |  |  |  |  |
| Chemical name | **Nominal concentration calculated (exp dilution serie)** | **Concentration in medium - Mass balance - in M** | **Dissolved concentration not bound - in M** | **Concentration bound to protein in M (Serum )** | **Concentration bound to lipid (Cell membrane) - in M** | **Concentration bound to plastic - in M** | **Concentration in headspace (evaporated) in M** | **LogP (as check point)** |
| Vincl | 4,20E-07 | 4,12E-07 | 2,50E-07 | 0 | 1,43E-07 | 1,84E-08 | 1,32E-13 | 3,1 |
| Vincl | 2,10E-07 | 2,06E-07 | 1,25E-07 | 0 | 7,16E-08 | 9,22E-09 | 6,59E-14 | 3,1 |
| Vincl | 1,10E-07 | 1,08E-07 | 6,55E-08 | 0 | 3,75E-08 | 4,83E-09 | 3,45E-14 | 3,1 |
| Vincl | 5,00E-08 | 4,90E-08 | 2,98E-08 | 0 | 1,70E-08 | 2,19E-09 | 1,57E-14 | 3,1 |
| Vincl | 5,31E-06 | 5,20E-06 | 3,16E-06 | 0 | 1,81E-06 | 2,33E-07 | 1,67E-12 | 3,1 |
| Vincl | 2,66E-06 | 2,61E-06 | 1,58E-06 | 0 | 9,06E-07 | 1,17E-07 | 8,35E-13 | 3,1 |
| Vincl | 1,33E-06 | 1,30E-06 | 7,92E-07 | 0 | 4,53E-07 | 5,84E-08 | 4,17E-13 | 3,1 |
| Vincl | 6,60E-07 | 6,47E-07 | 3,93E-07 | 0 | 2,25E-07 | 2,90E-08 | 2,07E-13 | 3,1 |
|  |  |  |  |  |  |  |  |  |
| Chemical name | **Nominal concentration calculated (exp dilution serie)** | **Concentration in medium - Mass balance - in M** | **Dissolved concentration not bound - in M** | **Concentration bound to protein in M (Serum)** | **Concentration bound to lipid (Cell membrane) in M** | **Concentration bound to plastic in M** | **Concentration in headspace (evaporated) in M** | **LogP (as check point)** |
| TCDD | 6,00E-07 | 5,71E-07 | 1,28E-11 | 0 | 5,65E-07 | 5,85E-09 | 1,81E-15 | 7,01 |
| TCDD | 3,00E-07 | 2,86E-07 | 6,40E-12 | 0 | 2,83E-07 | 2,93E-09 | 9,06E-16 | 7,01 |
| TCDD | 1,50E-07 | 1,43E-07 | 3,20E-12 | 0 | 1,41E-07 | 1,46E-09 | 4,53E-16 | 7,01 |
| TCDD | 8,00E-08 | 7,61E-08 | 1,71E-12 | 0 | 7,54E-08 | 7,80E-10 | 2,42E-16 | 7,01 |
| TCDD | 4,00E-08 | 3,81E-08 | 8,53E-13 | 0 | 3,77E-08 | 3,90E-10 | 1,21E-16 | 7,01 |
| TCDD | 2,00E-08 | 1,90E-08 | 4,26E-13 | 0 | 1,88E-08 | 1,95E-10 | 6,04E-17 | 7,01 |
| TCDD | 1,00E-08 | 9,52E-09 | 2,13E-13 | 0 | 9,42E-09 | 9,75E-11 | 3,02E-17 | 7,01 |
| TCDD | 5,00E-09 | 4,76E-09 | 1,07E-13 | 0 | 4,71E-09 | 4,88E-11 | 1,51E-17 | 7,01 |

**Reference**

Comenges JMZ, Joossens E, Benito JVS, Worth A, Paini A (2017) Theoretical and mathematical foundation of the Virtual Cell Based Assay - A review. Toxicol In Vitro 45(Pt 2):209-221 doi:10.1016/j.tiv.2016.07.013

Di Consiglio E, Pistollato F, Mendoza-De Gyves E, Bal-Price A, Testai E (2020) Integrating biokinetics and in vitro studies to evaluate developmental neurotoxicity induced by chlorpyrifos in human iPSC-derived neural stem cells undergoing differentiation towards neuronal and glial cells. Reprod Toxicol 98:174-188 doi:10.1016/j.reprotox.2020.09.010

Proença S, Escher BI, Fischer FC, et al. (2021) Effective exposure of chemicals in in vitro cell systems: A review of chemical distribution models. Toxicol In Vitro 73:105133 doi:10.1016/j.tiv.2021.105133

Proença S, Paini A, Joossens E, et al. (2019) Insights into in vitro biokinetics using Virtual Cell Based Assay simulations. Altex 36(3):447-461 doi:10.14573/altex.1812101

Zaldívar Comenges JM, Mennecozzi M, Macko P, Rodrigues R, Bouhifd M, Baraibar J (2011) A biology-based dynamic approach for the modelling of toxicity in cell assays: Part II: Models for cell population growth and toxicity. JRC Report EUR 24374 EN. Publications Office of the European Union, Luxembourg. doi:10.2788/61603.

Zaldívar Comenges JM, Mennecozzi M, Marcelino Rodrigues R, Bouhifd M (2010) A biology-based dynamic approach for the modelling of toxicity in cell-based assays. Part I: Fate modelling. JRC Report EUR 24374 EN. Publications Office of the European Union, Luxembourg. doi:10.2788/94002.
