# Supplementary material for: Combining in vitro assays and mathematical modelling to study developmental neurotoxicity induced by chemical mixtures
Source: Reprod Toxicol. 2021 Oct;105:101–19. doi: 10.1016/j.reprotox.2021.08.007 (PMC8522961; doi:10.1016/j.reprotox.2021.08.007)
Supplement: Supplementary file 3 [file mmc3.docx]

| **Supplementary Table S1.** BDE47, Ethanol, Vinclozolin and TCDD: Biological effects and concentrations found in human samples | | | |
| --- | --- | --- | --- |
| **Chemical name** | **Class** | **Main effects** | **Epidemiological data: concentrations found in human biological samples** |
| Polybrominated Diphenyl Ether 47 (BDE47)  (*Similar MoA*) | Organobromine compound, used as flame retardants, POP | One of the most pervasive PBDE congeners.  Impaired functional neuronal maturation and altered expression of neurodevelopmentally-relevant chromatin remodelers (Poston, Dunn et al. 2018).  Reduced neuronal and oligodendrocyte differentiation of human neural progenitor cells (Schreiber, Gassmann et al. 2010).  Perturbed calcium homeostasis (Gassmann, Schreiber et al. 2014).  In developing rats: interacted with PFOS inducing upregulation of BDNF in the cortex on PND 1 (synergistic effects); (antagonistic effects) on BDNF levels in the hippocampus on PND 14 (Wang, Liu et al. 2011).  Caused spatial memory deficits in perinatally exposed mice (Koenig, Lango et al. 2012).  Prenatal and childhood exposure to PBDEs (not only BDE-47) was found associated with poorer attention, fine motor coordination, and cognition in children (Eskenazi, Chevrier et al. 2013). | Breast milk:  - 2.03 ± 2.41 ng/g lipid (95^th^ percentile: 5.55 ng/g lipid) (Schlumpf, Kypke et al. 2010)  - 22.6 ng/g lipid (range 9.4–81.8) (Hartle, Cohen et al. 2018)  Maternal urine:  - 19 ng/g lipids (Woods, Lanphear et al. 2017)  Maternal blood:  - 0.24 ng/g lipid (range 0.22-0.26) (Dereumeaux, Saoudi et al. 2016)  - 37.78 μg/kg lipid-adjusted (95th Percentile) (Fisher, Arbuckle et al. 2016)  - 9.6 ± 15.3 pg/ml (Vafeiadi, Vrijheid et al. 2014)  Cord blood :  - 125 μg/kg lipid-adjusted (95^th^ percentile) (Fisher, Arbuckle et al. 2016) |
| Ethanol  (*Similar MoA*) | Industrial chemical | Decreases BDNF expression and/or impairs BDNF receptor (TrkB) capacity to transduce signals. Also, up-regulates BDNF in specific brain regions, and the signaling pathways associated with anxiety, addiction and homeostasis (Davis 2008).  Increased bdnf expression in the hippocampus of adult and aged offspring rats periconceptionally exposed to alcohol, which led to behavioural changes in adult and aged offspring (Lucia, Burgess et al. 2019).  Prenatal exposure to ethanol decreased hippocampal BDNF expression, leading to the disruption of hippocampal neurogenesis, apoptosis and decreased synaptic plasticity in adult rats (Yu, Shi et al. 2020).  Altered growth cone Ca^2+^ signaling, causing abnormal neuromorphogenesis in dissociated hippocampal cultures (Mah, Fleck et al. 2011).  Inhibited acetylcholine muscarinic receptor-stimulated phosphoinositide metabolism, as shown in neonatal (not adult) rats (Balduini and Costa 1990).  Suppressed phosphatidic acid formation by phospholipase D (PLD), leading to inhibition of astrocyte proliferation (Schatter, Jin et al. 2005); PLD is involved in neurite outgrowth (Kanaho, Funakoshi et al. 2009).  Suppressed expression of proliferator-activated receptor gamma co-activator 1-alpha (PGC-1α) (involved in the regulation of mitochondrial biogenesis and respiration, cellular antioxidant defence mechanism, and maintenance of neuronal integrity and function); impaired mitochondrial function, and enhanced cellular toxicity in cultured neuronal cell line and human foetal brain NSC-derived primary neurons;  decreased steady-state intracellular cAMP levels, inhibiting cAMP-response element binding protein (CREB) phosphorylation (regulator of PGC-1α gene transcription) (Liu, Liu et al. 2014).  Prenatal ethanol exposure caused multiple behavioral and cognitive attention deficits in rats, similar to effects observed in foetal alcohol syndrome and attentional deficit and hyperactivity disorders (Brys, Pupe et al. 2014, Oshiro, Beasley et al. 2014, Wang, Martin et al. 2020). | Maternal blood and breast milk:  - 1.05 g/L (after 1h ingestion of 0.6 g/kg ethanol) (Kesäniemi 1974)  Maternal blood:  - 42.1-473 mg/dL (at delivery) (Schaff, Moreno et al. 2019)  Infant blood:  - 38.4-246.5 mg/dL (Schaff, Moreno et al. 2019)  Breast milk:  - 0.32 g/L (after 1h ingestion of 0.3 g/kg ethanol), with brestfed infants expected to receive 0.5-3.3% of the mothers weight-adjusted dosage (Mennella and Beauchamp 1991) |
| Vinclozolin (Vincl)  (*Dissimilar MoA*) | pesticide (antiandrogenic) | Competitive antagonist of the androgen receptor;  Combined with other chemicals in an anti-androgenic mixture: was found to alter the expression of genes encoding for components of excitatory glutamatergic synapses, migration and pathfinding control, glutamatergic and GABAergic neurons, and genes linked with increased risk of ASD, as shown by qPCR in medial preoptic area and in ventromedial hypothalamus in perinatally exposed rats at PND 6 (Lichtensteiger, Bassetti-Gaille et al. 2015).  When administered to female Dutch-belted rabbits *(Oryctolagus cuniculus)* (10 mg/kg/d dietary), offspring showed fewer gonadotropin-releasing hormone expressing neurons and increased calbindin neurons in reproductive brain centres (Bisenius, Veeramachaneni et al. 2006).  Increased by 2-fold the level of gonadotrophin-releasing hormone in the mediobasal hypothalamus in prenatally exposed rabbits (Wadas, Hartshorn et al. 2010).  Can cause severe and widespread neuroendocrine disruptions in specific brain regions (e.g., hippocampus, amygdala, hypothalamus), leading to behavioural changes (e.g., cognitive deficits) in different species (León-Olea, Martyniuk et al. 2014). | Cord blood:  - 0.49-1.85 ng/ml (max level) at delivery (Wickerham, Lozoff et al. 2012) |
| 2,3,7,8-Tetrachlorodibenzo-p-dioxin (TCDD)  (*Dissimilar MoA*) | POP  (estrogenic) | Aryl-hydrocarbon receptor (AhR) agonist;  found to increase numbers of neural rosettes, and increase gene expression and protein levels of MAP2 and TH in human ESCs undergoing neuroectodermal commitment (Sarma, Nagano et al. 2019).  In PC12 neuronal cells: induced a time- and dose-dependent increase in nNOS expression, and high NO levels caused mitochondrial cytochrome c release, down-regulation of Bcl-2, increase of cleaved caspase-3 and apoptosis (Jiang, Duan et al. 2014).  In PC12 neuronal cells: at 100, 200, or 500 nM increased glucose-regulated protein (GRP78) and C/EBP homologous protein (CHOP) levels (hallmarks of endoplasmic reticulum stress) (Duan, Zhao et al. 2014).  Induced astrocyte reactivity (in vitro and in vivo) via phosphorylation of TGF-β-activated kinase 1 (TAK1, a regulator of NF-κB signalling) (Wan, Zhang et al. 2015).  Inhibited neurite outgrowth in differentiating human SH-SY5Y neuroblastoma cells (1-5 nM, for 2d) (Jung, Moon et al. 2009).  Inhibited sensory cortex function and downregulated expression of NMDA and AMPA receptor subunits in prenatally exposed rats (Hood, Woods et al. 2006).  Can cause rat thyroid morphologic and functional abnormalities through gestational and lactational exposure (Nishimura, Yonemoto et al. 2003).  Decreased composite motor and gross motor scores in boys exposed to TCDD (Tai, Nishijo et al. 2016). | Breast milk:  - 0.29 (<0.10-1.50) pg/g lipid (Schuhmacher, Mari et al. 2019)  - 0.019-0.71 pg/g lipid (Hernández, Pardo et al. 2020)  - 2.7 ± 1.4 pg/g lipid (Wang, Hang et al. 2019)  - 0.38 ± 0.51 ng/kg lipid (Rawn, Sadler et al. 2017)  - 2.2 ± 2.1 pg/g lipid (Boda, Nghi et al. 2018)  - 1.4 ± 2.3 pg/g lipid (Tai, Nishijo et al. 2016)  Cord blood or plasma:  - 1.49 pg/g lipid (Yu, Liu et al. 2019)  - 4.4 ± 2.4 pg/g lipid (Boda, Nghi et al. 2018)  Children serum:  - 2.9 (0.4–12.1) pg/g lipid (in 8–9 year-old boys) (Mínguez-Alarcón, Sergeyev et al. 2017) |
| **Studies cited in Supplementary Table S1:**  Balduini, W. and L. G. Costa (1990). "Developmental neurotoxicity of ethanol: in vitro inhibition of muscarinic receptor-stimulated phosphoinositide metabolism in brain from neonatal but not adult rats." Brain Res **512**(2): 248-252.  Bisenius, E. S., D. N. Veeramachaneni, G. E. Sammonds and S. Tobet (2006). "Sex differences and the development of the rabbit brain: effects of vinclozolin." Biol Reprod **75**(3): 469-476.  Boda, H., T. N. Nghi, M. Nishijo, P. N. Thao, P. T. Tai, H. Van Luong, T. H. Anh, Y. Morikawa, Y. Nishino and H. Nishijo (2018). "Prenatal dioxin exposure estimated from dioxins in breast milk and sex hormone levels in umbilical cord blood in Vietnamese newborn infants." Sci Total Environ **615**: 1312-1318.  Brys, I., S. Pupe and L. Bizarro (2014). "Attention, locomotor activity and developmental milestones in rats prenatally exposed to ethanol." Int J Dev Neurosci **38**: 161-168.  Davis, M. I. (2008). "Ethanol-BDNF interactions: still more questions than answers." Pharmacol Ther **118**(1): 36-57.  Dereumeaux, C., A. Saoudi, M. Pecheux, B. Berat, P. de Crouy-Chanel, C. Zaros, S. Brunel, C. Delamaire, A. le Tertre, A. Lefranc, S. Vandentorren and L. Guldner (2016). "Biomarkers of exposure to environmental contaminants in French pregnant women from the Elfe cohort in 2011." Environ Int **97**: 56-67.  Duan, Z., J. Zhao, X. Fan, C. Tang, L. Liang, X. Nie, J. Liu, Q. Wu and G. Xu (2014). "The PERK-eIF2α signaling pathway is involved in TCDD-induced ER stress in PC12 cells." Neurotoxicology **44**: 149-159.  Eskenazi, B., J. Chevrier, S. A. Rauch, K. Kogut, K. G. Harley, C. Johnson, C. Trujillo, A. Sjödin and A. Bradman (2013). "In utero and childhood polybrominated diphenyl ether (PBDE) exposures and neurodevelopment in the CHAMACOS study." Environ Health Perspect **121**(2): 257-262.  Fisher, M., T. E. Arbuckle, C. L. Liang, A. LeBlanc, E. Gaudreau, W. G. Foster, D. Haines, K. Davis and W. D. Fraser (2016). "Concentrations of persistent organic pollutants in maternal and cord blood from the maternal-infant research on environmental chemicals (MIREC) cohort study." Environ Health **15**(1): 59.  Gassmann, K., T. Schreiber, M. M. Dingemans, G. Krause, C. Roderigo, S. Giersiefer, J. Schuwald, M. Moors, K. Unfried, Å. Bergman, R. H. Westerink, C. R. Rose and E. Fritsche (2014). "BDE-47 and 6-OH-BDE-47 modulate calcium homeostasis in primary fetal human neural progenitor cells via ryanodine receptor-independent mechanisms." Arch Toxicol **88**(8): 1537-1548.  Hartle, J. C., R. S. Cohen, P. Sakamoto, D. B. Barr and S. L. Carmichael (2018). "Chemical Contaminants in Raw and Pasteurized Human Milk." J Hum Lact **34**(2): 340-349.  Hernández, C. S., O. Pardo, F. Corpas-Burgos, S. F. Fernández, A. López, C. Coscollà, M. Vento and V. Yusà (2020). "Biomonitoring of polychlorinated dibenzo-p-dioxins (PCDDs), polychlorinated dibenzofurans (PCDFs) and dioxin-like polychlorinated biphenyls (dl-PCBs) in human milk: Exposure and risk assessment for lactating mothers and breastfed children from Spain." Sci Total Environ **744**: 140710.  Hood, D. B., L. Woods, L. Brown, S. Johnson and F. F. Ebner (2006). "Gestational 2,3,7,8-tetrachlorodibenzo-p-dioxin exposure effects on sensory cortex function." Neurotoxicology **27**(6): 1032-1042.  Jiang, J., Z. Duan, X. Nie, H. Xi, A. Li, A. Guo, Q. Wu, S. Jiang, J. Zhao and G. Chen (2014). "Activation of neuronal nitric oxide synthase (nNOS) signaling pathway in 2,3,7,8-tetrachlorodibenzo-p-dioxin (TCDD)-induced neurotoxicity." Environ Toxicol Pharmacol **38**(1): 119-130.  Jung, J. E., J. Y. Moon, S. H. Ghil and B. S. Yoo (2009). "2,3,7,8-Tetrachlorodibenzo-p-dioxin (TCDD) inhibits neurite outgrowth in differentiating human SH-SY5Y neuroblastoma cells." Toxicol Lett **188**(2): 153-156.  Kanaho, Y., Y. Funakoshi and H. Hasegawa (2009). "Phospholipase D signalling and its involvement in neurite outgrowth." Biochim Biophys Acta **1791**(9): 898-904.  Kesäniemi, Y. A. (1974). "Ethanol and acetaldehyde in the milk and peripheral blood of lactating women after ethanol administration." J Obstet Gynaecol Br Commonw **81**(1): 84-86.  Koenig, C. M., J. Lango, I. N. Pessah and R. F. Berman (2012). "Maternal transfer of BDE-47 to offspring and neurobehavioral development in C57BL/6J mice." Neurotoxicol Teratol **34**(6): 571-580.  León-Olea, M., C. J. Martyniuk, E. F. Orlando, M. A. Ottinger, C. Rosenfeld, J. Wolstenholme and V. L. Trudeau (2014). "Current concepts in neuroendocrine disruption." Gen Comp Endocrinol **203**: 158-173.  Lichtensteiger, W., C. Bassetti-Gaille, O. Faass, M. Axelstad, J. Boberg, S. Christiansen, H. Rehrauer, J. K. Georgijevic, U. Hass, A. Kortenkamp and M. Schlumpf (2015). "Differential gene expression patterns in developing sexually dimorphic rat brain regions exposed to antiandrogenic, estrogenic, or complex endocrine disruptor mixtures: glutamatergic synapses as target." Endocrinology **156**(4): 1477-1493.  Liu, Z., Y. Liu, R. Gao, H. Li, T. Dunn, P. Wu, R. G. Smith, P. S. Sarkar and X. Fang (2014). "Ethanol suppresses PGC-1α expression by interfering with the cAMP-CREB pathway in neuronal cells." PLoS One **9**(8): e104247.  Lucia, D., D. Burgess, C. L. Cullen, E. S. Dorey, O. Rawashdeh and K. M. Moritz (2019). "Periconceptional maternal alcohol consumption leads to behavioural changes in adult and aged offspring and alters the expression of hippocampal genes associated with learning and memory and regulators of the epigenome." Behav Brain Res **362**: 249-257.  Mah, S. J., M. W. Fleck and T. A. Lindsley (2011). "Ethanol alters calcium signaling in axonal growth cones." Neuroscience **189**: 384-396.  Mennella, J. A. and G. K. Beauchamp (1991). "The transfer of alcohol to human milk. Effects on flavor and the infant's behavior." N Engl J Med **325**(14): 981-985.  Mínguez-Alarcón, L., O. Sergeyev, J. S. Burns, P. L. Williams, M. M. Lee, S. A. Korrick, L. Smigulina, B. Revich and R. Hauser (2017). "A Longitudinal Study of Peripubertal Serum Organochlorine Concentrations and Semen Parameters in Young Men: The Russian Children's Study." Environ Health Perspect **125**(3): 460-466.  Nishimura, N., J. Yonemoto, Y. Miyabara, M. Sato and C. Tohyama (2003). "Rat thyroid hyperplasia induced by gestational and lactational exposure to 2,3,7,8-tetrachlorodibenzo-p-dioxin." Endocrinology **144**(5): 2075-2083.  Oshiro, W. M., T. E. Beasley, K. L. McDaniel, M. M. Taylor, P. Evansky, V. C. Moser, M. E. Gilbert and P. J. Bushnell (2014). "Selective cognitive deficits in adult rats after prenatal exposure to inhaled ethanol." Neurotoxicol Teratol **45**: 44-58.  Poston, R. G., C. J. Dunn, P. Sarkar and R. N. Saha (2018). "Persistent 6-OH-BDE-47 exposure impairs functional neuronal maturation and alters expression of neurodevelopmentally-relevant chromatin remodelers." Environ Epigenet **4**(1): dvx020.  Rawn, D. F. K., A. R. Sadler, V. A. Casey, F. Breton, W. F. Sun, T. E. Arbuckle and W. D. Fraser (2017). "Dioxins/furans and PCBs in Canadian human milk: 2008-2011." Sci Total Environ **595**: 269-278.  Sarma, S. N., R. Nagano and S. Ohsako (2019). "Tyroxine Hydroxylase-Positive Neuronal Cell Population is Increased by Temporal Dioxin Exposure at Early Stage of Differentiation from Human Embryonic Stem Cells." Int J Mol Sci **20**(11).  Schaff, E., M. Moreno, K. Foster, M. G. Klug and L. Burd (2019). "What Do We Know About Prevalence and Management of Intoxicated Women During Labor and Delivery?" Glob Pediatr Health **6**: 2333794x19894799.  Schatter, B., S. Jin, K. Löffelholz and J. Klein (2005). "Cross-talk between phosphatidic acid and ceramide during ethanol-induced apoptosis in astrocytes." BMC Pharmacol **5**: 3.  Schlumpf, M., K. Kypke, M. Wittassek, J. Angerer, H. Mascher, D. Mascher, C. Vokt, M. Birchler and W. Lichtensteiger (2010). "Exposure patterns of UV filters, fragrances, parabens, phthalates, organochlor pesticides, PBDEs, and PCBs in human milk: correlation of UV filters with use of cosmetics." Chemosphere **81**(10): 1171-1183.  Schreiber, T., K. Gassmann, C. Götz, U. Hübenthal, M. Moors, G. Krause, H. F. Merk, N. H. Nguyen, T. S. Scanlan, J. Abel, C. R. Rose and E. Fritsche (2010). "Polybrominated diphenyl ethers induce developmental neurotoxicity in a human in vitro model: evidence for endocrine disruption." Environ Health Perspect **118**(4): 572-578.  Schuhmacher, M., M. Mari, M. Nadal and J. L. Domingo (2019). "Concentrations of dioxins and furans in breast milk of women living near a hazardous waste incinerator in Catalonia, Spain." Environ Int **125**: 334-341.  Tai, P. T., M. Nishijo, T. N. Nghi, H. Nakagawa, H. Van Luong, T. H. Anh and H. Nishijo (2016). "Effects of Perinatal Dioxin Exposure on Development of Children during the First 3 Years of Life." J Pediatr **175**: 159-166.e152.  Vafeiadi, M., M. Vrijheid, E. Fthenou, G. Chalkiadaki, P. Rantakokko, H. Kiviranta, S. A. Kyrtopoulos, L. Chatzi and M. Kogevinas (2014). "Persistent organic pollutants exposure during pregnancy, maternal gestational weight gain, and birth outcomes in the mother-child cohort in Crete, Greece (RHEA study)." Environ Int **64**: 116-123.  Wadas, B. C., C. A. Hartshorn, E. R. Aurand, J. S. Palmer, C. E. Roselli, M. L. Noel, A. C. Gore, D. N. Veeramachaneni and S. A. Tobet (2010). "Prenatal exposure to vinclozolin disrupts selective aspects of the gonadotrophin-releasing hormone neuronal system of the rabbit." J Neuroendocrinol **22**(6): 518-526.  Wan, C., Y. Zhang, J. Jiang, S. Jiang, X. Nie, A. Li, A. Guo and Q. Wu (2015). "Critical Role of TAK1-Dependent Nuclear Factor-κB Signaling in 2,3,7,8-Tetrachlorodibenzo-p-dioxin-induced Astrocyte Activation and Subsequent Neuronal Death." Neurochem Res **40**(6): 1220-1231.  Wang, F., W. Liu, Y. Jin, J. Dai, H. Zhao, Q. Xie, X. Liu, W. Yu and J. Ma (2011). "Interaction of PFOS and BDE-47 co-exposure on thyroid hormone levels and TH-related gene and protein expression in developing rat brains." Toxicol Sci **121**(2): 279-291.  Wang, R., C. D. Martin, A. L. Lei, K. A. Hausknecht, K. Ishiwari, J. B. Richards, S. Haj-Dahmane and R. Y. Shen (2020). "Prenatal Ethanol Exposure Leads to Attention Deficits in Both Male and Female Rats." Front Neurosci **14**: 12.  Wang, Z., J. G. Hang, H. Feng, L. L. Shi, J. J. Dong, B. Shen, T. Luo, R. M. Cai, L. J. Shen, T. Kido and X. L. Sun (2019). "Effects of perinatal dioxin exposure on development of children: a 3-year follow-up study of China cohort." Environ Sci Pollut Res Int **26**(20): 20780-20786.  Wickerham, E. L., B. Lozoff, J. Shao, N. Kaciroti, Y. Xia and J. D. Meeker (2012). "Reduced birth weight in relation to pesticide mixtures detected in cord blood of full-term infants." Environ Int **47**: 80-85.  Woods, M. M., B. P. Lanphear, J. M. Braun and L. C. McCandless (2017). "Gestational exposure to endocrine disrupting chemicals in relation to infant birth weight: a Bayesian analysis of the HOME Study." Environ Health **16**(1): 115.  Yu, D., X. Liu, X. Liu, W. Cao, X. Zhang, H. Tian, J. Wang, N. Xiong, S. Wen, Y. Wu, X. Sun and Y. Zhou (2019). "Polychlorinated Dibenzo-p-Dioxins, Polychlorinated Dibenzofurans, and Dioxin-Like Polychlorinated Biphenyls in Umbilical Cord Serum from Pregnant Women Living Near a Chemical Plant in Tianjin, China." Int J Environ Res Public Health **16**(12).  Yu, Y., Z. Shi, D. Xu, Y. Li, J. Qin, Z. Zhang and H. Wang (2020). "Prenatal ethanol exposure increases susceptibility to depression- and anxiety-like behavior in adult female offspring and its underlying mechanism." Reprod Toxicol **96**: 36-46. | | | |

| **Supplementary Table S2.** Summary of the main DNT effects induced by single chemicals administered at IC_20_, IC_5_ and IC_20_/100. In brackets, the lowest concentration eliciting a statistically significant alteration of at least one of the measured DNT features is indicated.  (BPA, CPF, Lead, Methyl-Hg, PCB138 and VA effects were described in Pistollato et al. 2020) | | | | | | | | | | |
| --- | --- | --- | --- | --- | --- | --- | --- | --- | --- | --- |
| **14 days** | **BPA** | **CPF** | **Lead** | **BDE47** | **EtOH** | **Methyl -Hg** | **PCB138** | **VA** | **Vincl** | **TCDD** |
| **BDNF** | ⇔  ⇓ ratio (IC_5_) | ⇑ BDNFt  ⇓ ratio (IC_20_) | ⇔ | ⇑ BDNFn (IC_20_/100) | ⇑ BDNFt (IC_5_) | ⇓ ratio (IC_20_) | ⇑ (IC_5_) | ⇑ BDNFn and ratio (IC_20_) | ⇑ BDNF (IC_20_/100) | ⇔ |
| **Neurite outgrowth** | ⇓ n. branch points (IC_5_) | ⇓ ⇔ | ⇔ | ⇑ neurite length & branch points  (IC_20_/100) | ⇑ neurite length (IC_20_) | ⇓ n. branch points (IC_20_) | ⇔ | ⇔ | ⇑ neurite length (IC_5_) | ⇑ neurite length (IC_20_/100) |
| **Synaptogenesis**  **(SYP, PSD95)** | ⇓ PSD95n (IC_5_) | ⇓ SYPn  ⇑ PSD95t (IC_5_) | ⇑ SYPt  ⇓ PSD95n (IC_20_/100) | ⇑ PSD95t  & SYPt (IC_5_) | ⇑ SYPt & PSD95t (IC_5_) | ⇓ SYPn (IC_5_) | ⇔ | ⇔ | ⇑ PSD95 ratio (IC_20_/100) | ⇑ PSD95t (IC_5_) |
| Abbreviations and symbols: t, total (cell body + neurite compartments); b, cell body compartment; n, neurite compartment; ratio, neurite-to-cell body ratio; ⇑, tendency towards increase; ⇓, tendency towards decrease; ⇔, no significant changes. | | | | | | | | | | |

**Supplementary Tables S3-S6.** BMD values calculated for each DNT endpoint and each individual chemical.

| ***Table S3*** | **Total BDNF** | | | |
| --- | --- | --- | --- | --- |
| **chemical** | **Best Model** | **Best BMD** | **Best BMDL** | **Best BMDU** |
| **BPA** | Power | 34.7238 | 31.2714 | 38.176 |
| **CFP** | Poly 2 | 11.7151 | 9.25353 | 15.8442 |
| **Lead** | Exp 2 | 1.87367 | 1.56809 | 2.37025 |
| **BDE47** | Exp 3 | 7.57079 | 6.16239 | 8.86543 |
| **EtOH** | Exp 2 | 242.144 | 193.695 | 330.548 |
| **Methyl-Hg** | Exp 3 | 0.321337 | 0.307307 | 0.332251 |
| **PCB138** | Exp 2 | 2.65338 | 2.38767 | 2.98547 |
| **VA** | Poly 2 | 257.677 | 197.831 | 310.248 |
| **Vincl** | Power | 142.257 | 92.5431 | 255.209 |
| **TCDD** | Poly 2 | 985.501 | 898.378 | 1182.11 |

| ***Table S4*** | **Neurite Length** | | | | **Branch Points/neurite** | | | | **N. Neurites/neuron** | | | |
| --- | --- | --- | --- | --- | --- | --- | --- | --- | --- | --- | --- | --- |
| **chemical** | Best Model | Best BMD | Best BMDL | Best BMDU | Best Model | Best BMD | Best BMDL | Best BMDU | Best Model | Best BMD | Best BMDL | Best BMDU |
| **BPA** | Linear | 21.6983 | 19.1325 | 25.0429 | Poly 2 | 72.965 | 68.2754 | 79.5805 | Power | 55.6289 | 51.2721 | 58.921 |
| **CFP** | Poly 2 | 60.77 | 48.98 | 180.16 | Power | 42.328 | 1.63E-07 | 371000 | Exp 2 | 46.9566 | 35.0446 | 69.9243 |
| **Lead** | Linear | 0.924882 | 0.812721 | 1.07224 | Linear | 1.40828 | 1.18245 | 1.74007 | Poly 2 | 6.72 | 3.61 | 71128.9 |
| **BDE47** | Linear | 41.236 | 19.9888 | 1844160 | Poly 2 | 11.9159 | 11.0995 | 12.8093 | Poly 2 | 13.1415 | 12.1448 | 14.5048 |
| **EtOH** | Poly 2 | 104.346 | 73.3226 | 456.923 | Poly 2 | 410.523 | 341.934 | 679.266 | Poly 2 | 502.193 | 389.941 | 1326.23 |
| **Methyl-Hg** | Exp 5 | 0.051044 | 0.037961 | 3316 | Exp 5 | 0.050423 | 0.040135 | 0.058273 | Exp 5 | 0.049375 | 0.036161 | 3316 |
| **PCB138** | Linear | 49.9303 | 17.47 | 884540 | Exp 2 | 26.553 | 13.3139 | 478.345 | Poly 2 | 9.74224 | 9.15026 | 10.4942 |
| **VA** | Hill | 133.642 | 110.228 | 163.2 | Hill | 133.078 | 107.46 | 158.762 | Exp 2 | 125.59 | 114.531 | 138.876 |
| **Vincl** | Poly 2 | 303.703 | 284.187 | 341.277 | Poly 2 | 281.978 | 262.253 | 309.96 | Exp 2 | 128.938 | 109.913 | 156.463 |
| **TCDD** | Power | 102993 | 205.567 | 8300000 | Exp 2 | 1850.85 | 1079.72 | 6936.09 | Poly 2 | 383.039 | 211.373 | 816.462 |

| ***Table S5*** | **Total SYP** | | | | **Total PSD95** | | | | **Synapses** | | | |
| --- | --- | --- | --- | --- | --- | --- | --- | --- | --- | --- | --- | --- |
| **chemical** | Best Model | Best BMD | Best BMDL | Best BMDU | Best Model | Best BMD | Best BMDL | Best BMDU | Best Model | Best BMD | Best BMDL | Best BMDU |
| **BPA** | Linear | 21.1416 | 18.3013 | 25.0024 | Power | 25.7048 | 20.2721 | 31.6176 | Linear | 38.6334 | 30.9055 | 51.4938 |
| **CFP** | Exp 2 | 177.24 | 64.9554 | 371000 | Hill | 22.8465 | 20.604 | 13727000 | Poly 2 | 16.9471 | 13.5345 | 20.4833 |
| **Lead** | Power | 0.554752 | 0.255614 | 0.978329 | Power | 0.583324 | 0.228896 | 1.14368 | Linear | 1.28688 | 1.07875 | 1.59352 |
| **BDE47** | Power | 9.8667 | 8.22579 | 11.2846 | Exp 2 | 13.1578 | 9.89604 | 19.7297 | Poly 2 | 6.50253 | 5.03865 | 7.87897 |
| **EtOH** | Linear | 249.625 | 184.578 | 385.377 | Poly 2 | 234.608 | 213.358 | 255.13 | Linear | 344.301 | 232.797 | 660.713 |
| **Methyl-Hg** | Power | 0.336387 | 0.327169 | 0.378722 | Poly 2 | 0.157031 | 0.094241 | 0.789495 | Power | 0.333075 | 0.29427 | 0.49026 |
| **PCB138** | Poly 2 | 12.8775 | 11.444 | 16.3892 | Power | 0.544732 | 0.155543 | 1.51296 | Poly 2 | 8.96376 | 8.1606 | 9.85511 |
| **VA** | Power | 509.299 | 368.709 | 525.142 | Power | 110.733 | 3.98817 | 6275.22 | Exp 2 | 286.793 | 244.671 | 349.185 |
| **Vincl** | Exp 2 | 117.4 | 101.869 | 139.004 | Exp 2 | 75.1959 | 66.9029 | 85.7927 | Power | 386.565 | 134.007 | 3217.33 |
| **TCDD** | Poly 2 | 950.626 | 883.259 | 1066.27 | Exp 4 | 697.273 | 215.587 | 8300000 | Poly 2 | 978.373 | 902.033 | 1123.2 |

| ***Table S6*** | **%GFAP** | | | | **% β-III-Tubulin** | | | |
| --- | --- | --- | --- | --- | --- | --- | --- | --- |
| **chemical** | Best Model | Best BMD | Best BMDL | Best BMDU | Best Model | Best BMD | Best BMDL | Best BMDU |
| **BPA** | Linear | 25.007 | 20.5909 | 31.7972 | Exp 2 | 128.469 | 77.6085 | 414.171 |
| **CFP** | Linear | 13.3903 | 11.3891 | 16.2326 | Poly 2 | 8.95409 | 7.37515 | 11.3935 |
| **Lead** | Power | 0.247276 | 2.48E-07 | 1.06892 | Poly 2 | 4.23177 | 3.48625 | 7.74494 |
| **BDE47** | Linear | 25.8977 | 10.8432 | 720674 | Poly 2 | 11.1216 | 10.1815 | 12.0455 |
| **EtOH** | Linear | 120.58 | 97.8032 | 157.027 | Exp 2 | 436.568 | 285.422 | 878.37 |
| **Methyl-Hg** | Exp 3 | 0.286406 | 0.240915 | 0.306885 | Poly 2 | 0.321012 | 0.29725 | 0.344859 |
| **PCB138** | Exp 2 | 2.78203 | 2.54221 | 3.07197 | Power | 4.31838 | 3.34495 | 5.37669 |
| **VA** | Poly 2 | 277.186 | 226.763 | 319.692 | Poly 2 | 370.683 | 326.002 | 405.099 |
| **Vincl** | Exp 2 | 88.1074 | 80.6959 | 97.0674 | Poly 2 | 116.673 | 75.6958 | 157.591 |
| **TCDD** | Poly 2 | 1117.48 | 993.404 | 1477.32 | Linear | 45378.2 | 2454.41 | 6.89E+09 |
